# Research space telescope deployable mechanism variable diameter internal drive device elastic collision contact characteristics in the inter-stage transition

**DOI:** 10.1038/s41598-023-49992-4

**Published:** 2023-12-16

**Authors:** Yingjun Guan, Weiqi Huang, Hao Wang, Huanquan Lu, Huisheng Yang

**Affiliations:** 1 College of Electrical and Mechanical Engineering, Changchun University of Technology, Changchun, 130012 Jilin China; 2 Changchun Institute of Optics and Precision Mechanics and Physics, Chinese Academy of Sciences, Changchun, 130033 Jilin China

**Keywords:** Engineering, Mechanical engineering

## Abstract

To address the problem of elastic contact discrepancies between a variable-diameter internal drive device and a non-continuous surface during the transition, caused by the vibrations resulting from elastic collision impact when the motion speed of the elastic body increases, the following steps were taken. First, we established models for elastic collision, impact, and vibration during the inter-stage transition to analyze how motion speed and preload affect the elastic contact characteristics between the two components. Subsequently, we employed the finite element method to further analyze the elastic contact state, using identical loads but varying motion speeds and radial preloads as boundary conditions. Finally, an experimental prototype was developed to validate the elastic contact state of the elastic body during the inter-stage transition. The results indicated that the amplitude of elastic body vibration increased with higher motion speed, while it decreased with higher radial preload. Therefore, it is necessary to adjust radial preload at different times to ensure effective elastic contact between the elastic body and the non-continuous surface during the inter-stage transition. This approach not only enhances deployment speed but also guarantees the stability of the inter-stage transition.

## Introduction

At the current stage of development, the research and development of large-aperture observation equipment are facing a bottleneck due to the increasing demand from various countries for observing distant outer space. The application of the reducer internal drive device has effectively resolved the challenge of extending the observation equipment's distance and stiffness within the space split telescope deployable mechanism. However, it has given rise to another critical issue: ensuring stability during the inter-stage transition while increasing the unfolding speed. Throughout the deployment process, the device forms a non-continuous inner surface within the sleeve, and it employs a rubber wheel (elastomer) to travel along the inner wall of the sleeve, which enables the spatial expansion mechanism. This mechanism is achieved by driving the rubber wheel and applying radial preload to gradually unfold and retract the stages. Nevertheless, during the extension and retraction phases, the rubber wheel encounters the need to traverse the discontinuous step surface between the stages. At lower speeds, the interaction merely incurs internal energy loss. However, as the speed increases, the rubber wheel, being an elastomer, experiences elastic collision impacts with the discontinuous step surface. These external impacts result in changes to the vibration amplitude of the elastomer, thus affecting the contact state between the rigid, non-continuous surface and the elastomer, ultimately reducing the stability of the inter-stage transition.

Over the past decade, extensive research has delved into the mechanical interactions between elastic and rigid materials. Notably, Colin Thornton^1^ and his contemporaries established equivalent spring models by simulating the impact of an elastic sphere against a rigid inclined surface during collisions. They conducted a comparative analysis of the rebound motion using various contact models, exploring both linear and nonlinear angles. D. Gugan^2^ furthered this investigation by measuring the contact area and contact duration when a sphere impacted a rigid body, focusing on the impact velocity as a variable. The outcomes corroborated the alignment of contact area and contact duration with Hertzian elastic collision theory. A.B. Stevens^3^ and a team of researchers developed a collision prediction model to simulate the impact recovery coefficient and collision duration of a soft sphere in contact with a rigid body surface. Empirical testing confirmed the adherence of the collision of a soft sphere to elastic collision theory, with the impact recovery coefficient^4^ and collision duration decreasing as the speed increased. Yury A. Rossikhin^5^ and colleagues investigated the collision of viscoelastic bodies using collision fluctuation theory. Their examination of the collision of two elastic spheres revealed that the contact domain solution could be obtained through a modified Hertzian contact model that incorporates the elastic body's Young's modulus and Poisson's ratio. In this modified contact model, instanton model^6^ operators were incorporated. Yury A. Rossikhin^7^ extended this work by solving the modified Hertzian contact model for models inside and outside the contact domain using conventional and fractional order derivatives, respectively. Experimental results highlighted that the fractional order derivative model could establish the relationship between elastomer deformation and the Hertzian contact model more efficiently, albeit with a higher computational cost. Concerning the intrinsic model of elastomers, Huanying Xu^8^ utilized the fractional derivative model to predictively describe the time-varying creep nodes of the intrinsic model and further fitted the best parameters. Experimental verification confirmed the effectiveness of the fractional derivative model for viscoelastic models. Adel Tayeb^9^ employed rubber material as an elastomer and constructed a three-dimensional elastic model to depict the dynamic response of rubber-like materials. They conducted least-squares identification of nodal parameters for the multiple-integral viscoelastic model and the BIIR(Brominated Isobutylene-Isoprene Rubbe) material model in both quasi-static and dynamic scenarios. These nodal parameters were then applied to predict the strain of rubber-like materials in collisions with rigid bodies. Maia M. Svanadze^10^ employed the linear Kelvin-V material of the double-hole viscoelastic theory to derive solutions for quasi-static and steady-state equations. Primitive functions were utilized to construct the solutions for the set of equations. In the realm of impact on elastic materials, Zhang Dongmei^11^ and colleagues explored the impact between bolts and elastic washers. They established an impact model based on collision theory and experimentally verified the strain data of elastic washers in impact cases. Amin Khodadadi^12^ and colleagues examined the response of rubber sheets under increased velocity impact loads. They developed an impact response model for elastic materials subjected to impact and observed the attenuation of the internal impact stress wave in rubber after increased velocity through simulation and experimental analysis. Md Fazlay Rabbi^13^ and colleagues established a linear physics-based model to study the one-dimensional impact problem of viscoelastic materials. They employed a Laplace transform-based analytical method to solve the impact problem. R. Springhett^14^ utilized articular cartilage as a viscoelastic material in elastic impact modeling. They observed that the tissue's hysteresis increased with impact velocity and introduced the Kelvin-Voigt relaxation function. Experimental results demonstrated significant improvements in describing both the stress–strain response and energy dissipation. Stelian Alaci^15^ and colleagues provided an analytical solution for the viscoelastic collision of two spherical centers. They divided the contact cycle into two phases: compression and recovery, bounded by the moment corresponding to the maximum deformation. The system's motion was described by a nonlinear Hunt-Crossley equation, which exhibited the advantage of hysteresis loop origin closure. They further simplified the equation to obtain the final viscoelastic impact model.

In the realm of elastomeric vibrations, Marco Amabili^16^ delved into the nonlinear vibrations of a viscoelastic rectangular thin plate subjected to normal simple harmonic excitation near the lowest resonance spectrum. In this study, the von Kármán nonlinear strain–displacement relation was employed, and the presence of geometric defects was considered. The material was characterized as a Kelvin-Voigt viscoelastic solid, encompassing all nonlinear terms. This investigation also examined the frequency response variation concerning the delay time parameter and the impact of geometric defects. Shifting our focus to another facet of the subject, A. Papangelo^17^ explored a spherical oscillator excited by a moving viscoelastic half-space. The study involved the calculation of frictional behavior and nonlinear normal contact stiffness through numerical simulations employing boundary elements. This exploration varied the substrate velocity and indentation depth to derive insights. The acquired findings were subsequently utilized in numerical simulations of dynamic interactions, culminating in the development of a comprehensive viscoelastic material vibration model. Przemyslaw Wielentejczyk^18^ and colleagues addressed the problem of geometrically nonlinear steady-state vibrations in an elastic beam under impact loading. They harnessed Von Karman theory to portray the geometrically nonlinear effects brought about by elastic beam deformation. The steady-state solution of the vibration equation was rigorously validated through a series of simulations. In a distinctive approach to the nonlinear vibration problem of elastic beams, Ehsan Loghman^19^ and collaborators harnessed the fractional-order viscoelastic functional gradient function. This innovative method considered the microscale effect by incorporating the modified couple stress theory (MCST) and relied on a fractional-order Kelvin-Voigt viscoelastic model^20^. The equations of motion were formulated using Hamilton's principle, resulting in the establishment of a novel discontinuous vibration model specifically designed for viscoelastic materials.

The concept of the carrier (variable diameter internal drive device) under study in this article is introduced for the first time and applied to the telescopic deployable mechanism of space telescopes. Compared to traditional driving and tensioning devices in the space telescope's telescopic deployable mechanism, this innovative approach offers several advantages, including high deployment accuracy, significant stiffness, and extended deployment distance. However, as research progresses^21,22^, it becomes apparent that, owing to the nested configuration of the sleeve, a discontinuous stepped surface emerges during the deployment process. When the variable diameter internal drive device moves within the sleeve, the elastic material comes into contact with and collides against this discontinuous surface. This interaction impacts the stability and efficiency of the space telescope's deployment. Consequently, it is essential to delve further into the elastic collision contact characteristics of the variable diameter internal drive device during the inter-stage transition phase. The research conducted by the scholars mentioned above regarding the mechanical interactions between elastic and rigid materials has uncovered a notable research gap. This gap pertains to the contact behavior between elastomers and rigid bodies on discontinuous surfaces. More precisely, there is a limited understanding of how the impact-induced vibrations, arising from their collisions, influence the contact state. Additionally, there's a need to explore methods for minimizing this impact to improve the stability of the inter-stage transition, especially when increasing the deployment rate of the deployable mechanism.

This paper addresses this issue by introducing an intrinsic model of elastic material based on a three-parameter relationship and integrating it into the collision model. Since the contact between the elastomer and the discontinuous surface of the rigid body involves instantaneous rolling contact, it is essential to incorporate both normal contact load and tangential friction load into the collision model. This integration enables the establishment of a stress model for positive and negative waves within the structure through the impact model. Subsequently, these positive and negative waves are transformed into vibration waves, allowing for the analysis of vibration wave propagation within the structure and the determination of the elastomer's vibration amplitude. The validity of the developed elastic collision impact vibration model is then confirmed through finite element analysis and prototype testing.

The following sections structure the remainder of this paper. In Section "Analysis of inter-stage transition state in variable diameter internal drive device", we investigate the transition state during the inter-stage transition of the spatially deployable mechanism, analyzing elastomer motion at both low and increased velocities. Section "Method" develops a model to depict the mechanical properties of collision, impact, and vibration between the elastomer and the rigid body throughout the inter-stage transition while also investigating the factors influencing the inter-stage contact state. Section "Finite element analysis of elastic body during inter-stage transition" employs the finite element method to simulate the inter-stage transition stage, conducting a comprehensive analysis that incorporates the elastic collision, display dynamics, and vibration modules. In Section "Experimental and discussion", a 1:1 prototype model is constructed to conduct inter-stage transition tests on the elastomer using the control variable method. Finally, Section "Conclusions" offers a summary of the entire paper and outlines the scope of future work.

## Analysis of inter-stage transition state in variable diameter internal drive device

The spatially deployable mechanism comprises a variety of spreading and retracting structures that may vary in dimensionality. One-dimensional spreading structures, like the sleeve extension arm, offer high stiffness and precision. The sleeve extension arm's spreading function is predominantly dependent on the activation of the pre-tensioning device. Previous research has employed screw, rope, and tension integration systems to achieve the desired spreading function. However, as the demand for observation continues to grow, limitations regarding spreading distance and stiffness have become increasingly evident.

**Figure 1:**
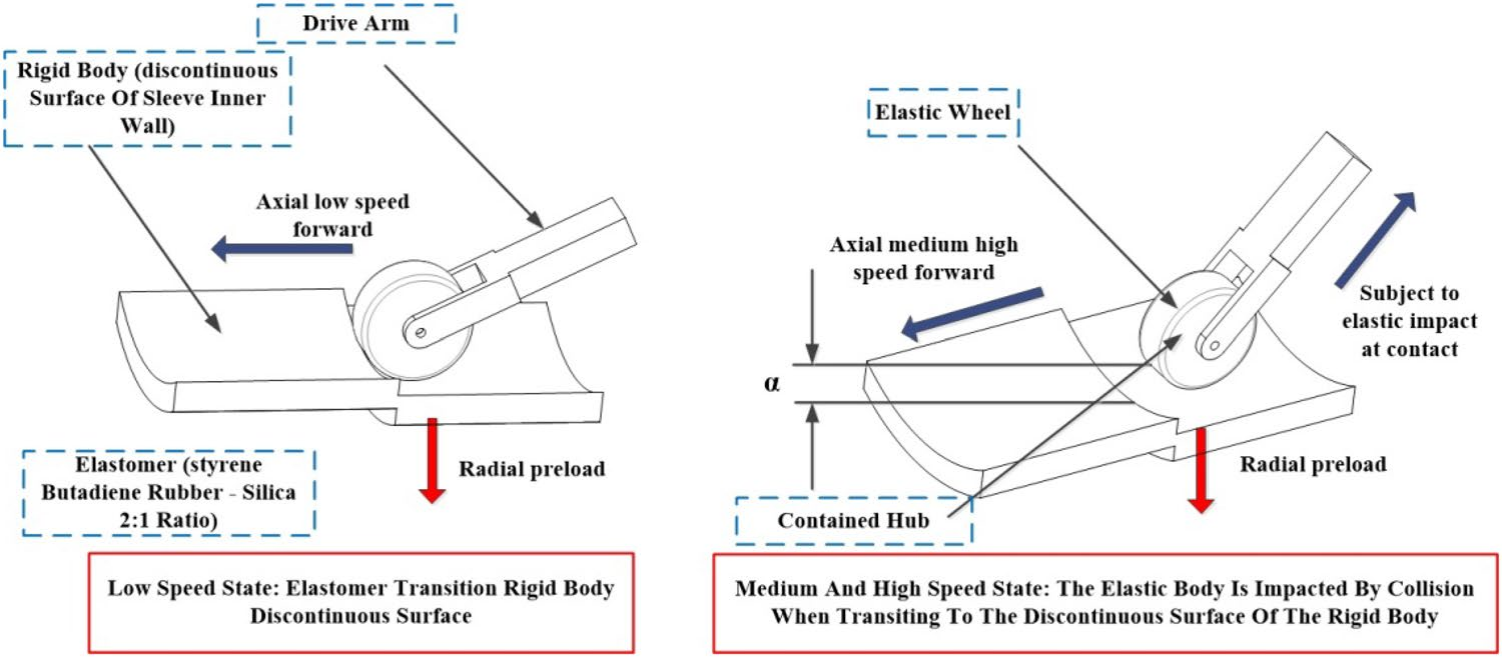
Axial climbing propulsion state during inter-stage transition (left: low speed, right: increased speed).

In an effort to overcome the limitations of conventional drive preload structures, the implementation of a reducer internal drive device has been employed to facilitate the spreading and retracting function of the sleeve extension arm. However, further investigation has uncovered a specific issue concerning the non-continuous nature of the inner wall of the sleeve. This discontinuous, step-like structure within the reducer internal drive device introduces resistance to the rubber wheel during the inter-stage transition. As the speed of the rubber wheel increases, it leads to high-intensity collisions, impacts, and vibrations at the contact points. Consequently, these collisions, impacts, and vibrations have the potential to induce the retreat or slippage of the reducer internal drive during axial climbing propulsion in the inter-stage transition stage, as illustrated in Fig. 1. In this context, our analysis stems from prior research on the flexible multibody dynamics of the variable-diameter internal drive device and the investigation of elastic frictional contact characteristics during inter-stage transition phases^21,22^. This comprehensive study is complemented by the calculation of the fixed frequency of the elastic material (Emulsion-polymerized styrene butadiene rubber). The ultimate result of our calculations discloses a minimum velocity of 2.9741 mm/s, rounded for computational convenience to approximately 3 mm/s. This identified minimum velocity not only imparts vibrational response capabilities to the elastic body but also aligns closely with the inherent frequency of the elastic body.

The drive shaft rotates the hub, which subsequently drives the elastomer wrapped around it to rotate. Simultaneously, the drive arm applies radial preload to the elastomer, hub, and drive shaft. At lower speeds, the interaction of the elastomer with the discontinuous step surface on the rigid body is primarily influenced by its internal potential energy. However, this effect remains negligible during low-speed axial climbing, as the contact state between the elastomer and the rigid body remains stable. With the increase in speed, the interaction between the elastomer and the non-continuous surface of the rigid body is now influenced by both the kinetic and potential energy of the elastomer. This results in a motion trend in the normal direction at the contact point, brought about by collisions, impacts, and vibrations between the two components. Consequently, a gap in distance (denoted as '$\alpha$') is created, which further impacts the contact state between the elastomer and the rigid body.

Based on our preliminary investigations, we have established that, when subjected to top loads, the variable-diameter internal drive device necessitates a calculated minimum radial preload force of approximately 100N to operate effectively within the sleeve. This specified force of 100N corresponds to one-third of the complete structure of the variable-diameter internal drive device and is applied as a radial preload force to the inner wall of the sleeve. Further elucidation on the symmetry of the variable-diameter internal drive device structure will be provided in the finite element analysis section. Furthermore, an empirical observation indicates that, with an increase in rolling speed under a consistent radial preload force between the elastic body and the non-continuous surface rigid body, the collision contact gap gradually expands proportionally to the rising speed. Undoubtedly, investigating the interplay between these factors will constitute a central focus in the subsequent phases of our research.

Regardless of the speed of motion, be it low or high, the driving arm imparts a radial load onto the elastic wheel. However, at lower speeds, the manifestation of internal potential and kinetic energy feedback is less prominent. The applied radial load induces a smaller gap, swiftly compensated by the motion trend of the radial load. With an escalation in motion speed, the elastic potential and kinetic energy of the wheel further intensify. When the radial load maintains its previous magnitude, the motion trends between the two components cannot be counteracted, leading to an enlarged gap. It is noteworthy that the driving arm retains mechanical energy on different surfaces. Nevertheless, variations in sleeve diameter occur, and when the speed of the variable-diameter internal drive device remains constant, the radial preload load fluctuates in tandem with the changes in sleeve diameter.

**Figure 2:**
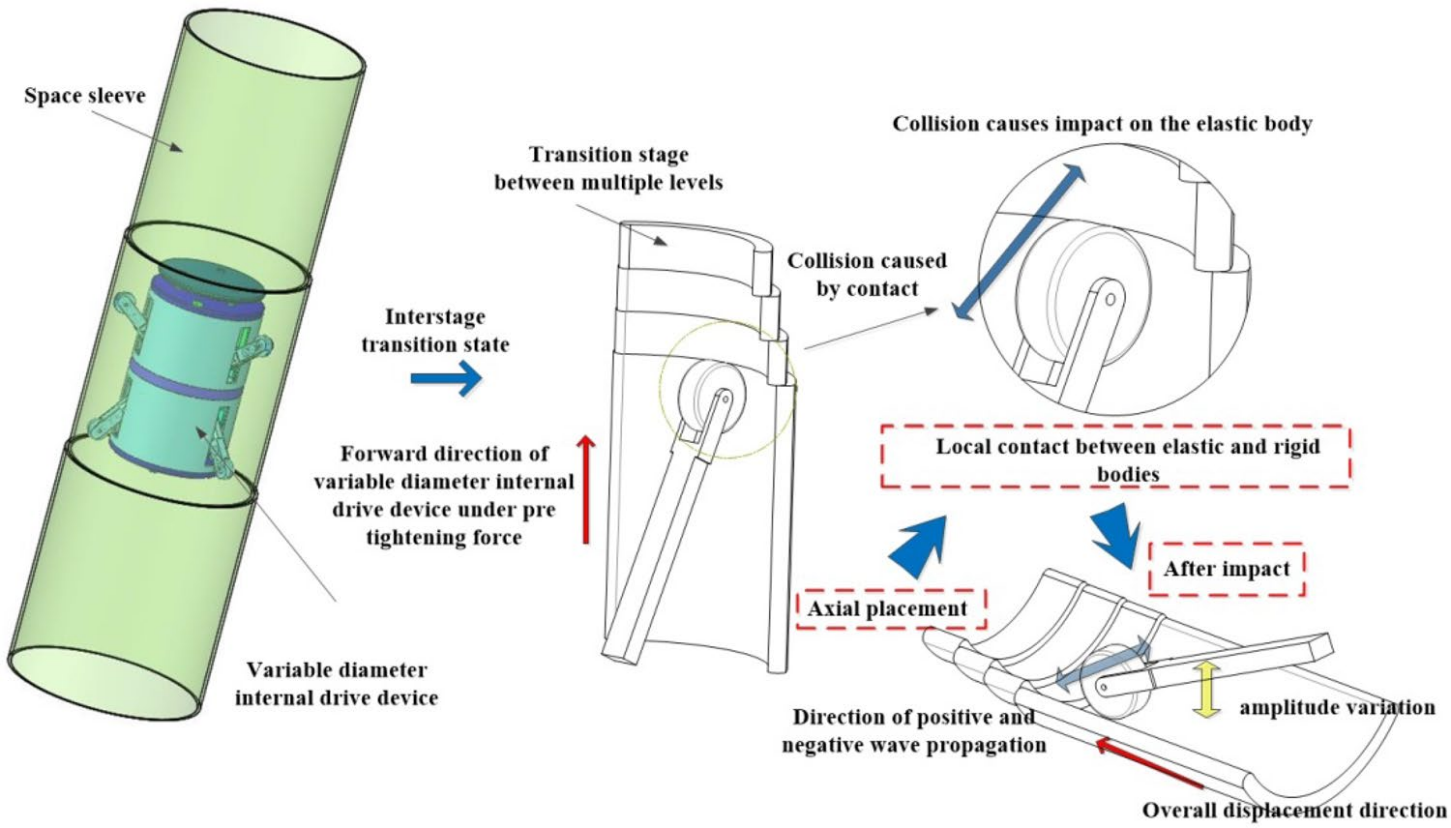
Schematic of structural collision impact and vibration.

Hence, it becomes imperative to investigate the mechanical characteristics of the rubber wheel in its interaction with the non-continuous surface during the inter-stage transition phase, particularly as it pertains to its behavior at increased speeds (illustrated in Fig. 2). This study primarily focuses on assessing how the external excitation resulting from impact loads affects the amplitude of vibration in the elastomer during its collision with the rigid body. The changes in vibration amplitude, in turn, have implications for the contact between the elastomer and the rigid body, which plays a pivotal role in determining whether the variable-diameter internal drive can smoothly transition at elevated speeds during the inter-stage transition. Additionally, it is of great significance to take into account the conditions involving axial gravitational loads, radial preloading forces, and transient frictional interactions experienced by the rubber wheel in the course of the inter-stage transition.

## Method

### Elastic collision model

In order to investigate the contact condition between the elastomer and the rigid body at higher motion speeds, we establish an elastic collision model between the elastomer (rubber wheel) and the rigid body (sleeve's discontinuous step surface). This model involves an analysis of collision forces and the transmission of positive and negative waves generated by the impact load within the elastomer. These positive and negative waves act as external excitations for calculating the elastomer's vibration amplitude during its collision with the rigid body. Subsequently, we derive the contact condition between the two.

#### Elastic material constitutive factors

**Figure 3:**
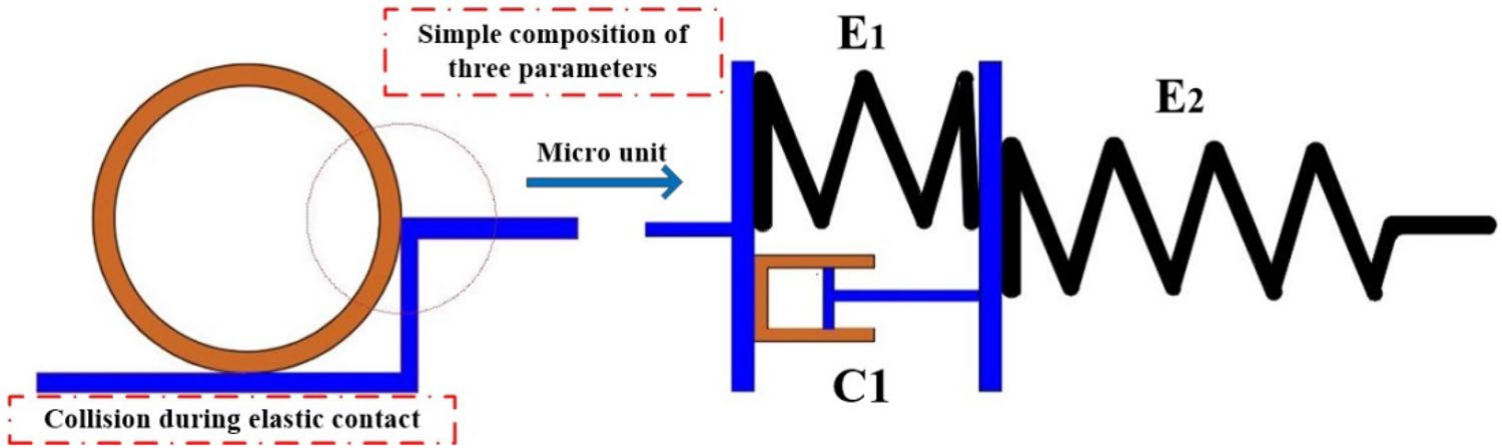
Three-parameter model diagram.

First, we establish the intrinsic model for the elastomeric rubber material. It's widely recognized that the Kelvin model falls short in representing the material's transient elasticity. In practice, many materials exhibit transient elasticity, which is especially significant during collision. Therefore, we employ the three-parameter model illustrated in Fig. 3.

Define respectively $\varepsilon_{1} ,\varepsilon_{2} ,\sigma ,\varepsilon$ as the strain of the Kelvin model, the strain of the spring in series with it, the total model stress and the total model strain, and then the following relationship exists:

1 $$ \left\{ \begin{gathered} \varepsilon = \varepsilon_{1} + \varepsilon_{2} \hfill \\ \sigma = E_{1} \varepsilon_{1} + C_{1} \dot{\varepsilon }_{1} \hfill \\ \sigma = E_{2} \varepsilon_{2} \hfill \\ \end{gathered} \right. $$

$E_{1}$, $E_{2}$ are spring 1 and spring 2; $C_{1}$ is the damping. Laplace transformations are done on the above three equations:

2 $$ \left\{ \begin{gathered} \overline{\varepsilon } = \overline{\varepsilon }_{1} + \overline{\varepsilon }_{2} \hfill \\ \overline{\sigma } = \left( {E_{1} + C_{1} s} \right)\overline{\varepsilon }_{1} \hfill \\ \overline{\sigma } = E_{2} \overline{\varepsilon }_{2} \hfill \\ \end{gathered} \right. $$

From the latter two equations $\overline{\varepsilon }_{1} ,\overline{\varepsilon }_{2}$ the standard form of the three-parameter model is obtained by back-substituting and then doing the Laplace inversion:

3 $$ \sigma + p_{1} \dot{\sigma } = q_{0} \varepsilon + q_{1} \dot{\varepsilon } $$

Among them:

4 $$ q_{0} = \frac{{E_{1} E_{2} }}{{E_{1} + E_{2} }},p_{1} = \frac{{C_{1} }}{{E_{1} + E_{2} }},q_{1} = \frac{{C_{1} E_{2} }}{{E_{1} + E_{2} }} $$

Sudden application of stress at $t = 0$ (near collision scenario):

5 $$ \sigma \left( t \right) = \sigma_{0} H\left( t \right) $$

In the domain of the Laplace transform:

6 $$ \overline{\sigma } = \sigma_{0} /s $$

7 $$ \overline{{\dot{\sigma }}} = s\overline{\sigma } = \sigma_{0} $$

Substituting (7) into the present constitutive equation:

8 $$ \sigma_{0} /s + p_{1} \sigma_{0} = q_{0} \overline{\varepsilon } + q_{1} s\overline{\varepsilon } $$

And then do the Laplace inversion to get^23^:

9 $$ \varepsilon \left( t \right) = \frac{{\sigma_{0} }}{{E_{2} }} + \frac{{\sigma_{0} }}{{E_{1} }}\left( {1 - e^{{ - E_{1} t/c_{1} }} } \right) $$

The slow decrease in relaxation stress with time can be expressed as:

10 $$ \sigma \left( t \right) = E_{2} \varepsilon_{0} - \frac{{E_{2}^{2} \varepsilon_{0} }}{{E_{1} + E_{2} }}\left( {1 - e^{{ - t/p_{2} }} } \right) $$

Intrinsic relations for viscoelastic substances under the Laplace transform:

11 $$ \overline{P}\left( s \right)\overline{\sigma }\left( s \right) = \overline{Q}\left( s \right)\overline{\varepsilon }\left( s \right) $$

Among them:

12 $$ \overline{P}\left( s \right) = \mathop \sum \limits_{k = 0}^{m} p_{k} s^{k} ,\overline{Q}\left( s \right) = \mathop \sum \limits_{k = 0}^{m} q_{k} s^{k} $$

Written in the form of an elastic principal structure relationship:

13 $$ \overline{\sigma }\left( s \right) = \frac{{\overline{Q}\left( s \right)}}{{\overline{P}\left( s \right)}}\overline{\varepsilon }\left( s \right),\overline{\varepsilon }\left( s \right) = \frac{{\overline{P}\left( s \right)}}{{\overline{Q}\left( s \right)}}\overline{\sigma }\left( s \right) = \overline{E}\left( s \right)\overline{\sigma }\left( s \right) $$

**Figure 4:**
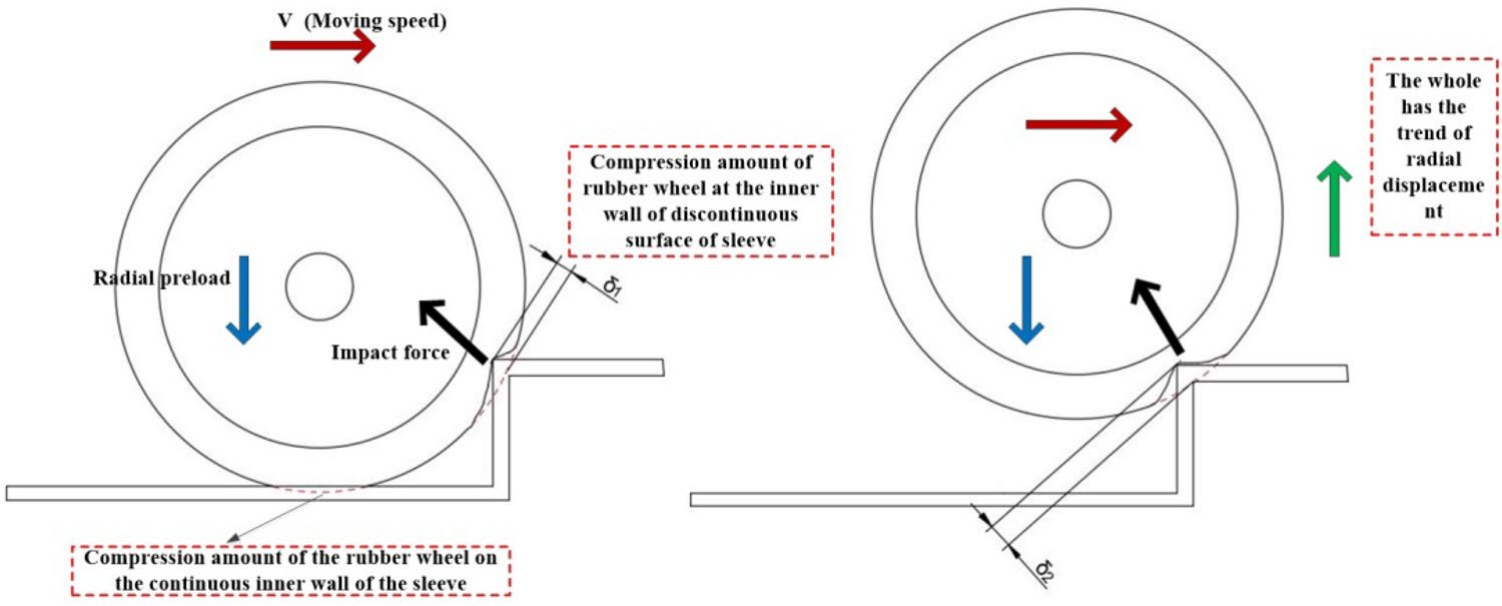
Schematic diagram of collision.

Through the linear elastic constitutive equations of viscoelastic materials and the theory of contact mechanics of viscoelastic materials, the collision force after a collision between two rigid and flexible objects is calculated as follows^24^:

14 $$ F = D\left( {A_{{\text{i}}} ,R_{i} ,l_{i} } \right) \cdot E^{*} \cdot \delta^{\alpha } \cdot V $$

where $D\left( {A_{{\text{i}}} ,R_{i} ,l_{i} } \right)$, The symbol 'i' denotes 'instantaneous.' A_instantaneous_ signifies the instantaneous contact area between two surfaces, while R_instantaneous_ represents the radius of curvature of the instantaneous contact surface. Additionally, L_instantaneous_ corresponds to the thickness of the elastic body during instantaneous contact. Parameters under the conditions of the contact area of the two object in collision, the radius of curvature of the contact surface, etc. is considered.$\delta$ is the intrusion displacement of the colliding two, at this time, is the compression intrusion depth $d$ of the viscoelastic material, which is consistent with the indentation depth d of the latter equation.$\alpha$ Parameters related to the contact properties of the interface.$E^{*}$ is the equivalent modulus of elasticity,$E^{*} = \sigma E_{1} /\varepsilon = \left( {E_{2} \varepsilon_{2} E_{1} } \right)/\left( {\varepsilon_{1} + \varepsilon_{2} } \right)$.Among them, $\sigma$ represents the total stress of the three parameter model, $\varepsilon$ represents the total strain of the three parameter model, and $E_{1}$ and $E_{2}$ represent the elastic modulus of the three parameter model. ${\text{V}}$ is the collision velocity. As shown in Fig. 4 for the collision of an elastomer with a rigid body.

The Laplace transformation of the above equation yields:

15 $$ \overline{F} = \overline{D}\left( {A_{i} ,R_{i} ,l_{i} } \right) \cdot \overline{E}^{*} \cdot \overline{\delta }^{\alpha } \cdot \overline{V} $$

Since $D\left( {A_{{\text{i}}} ,R_{i} ,l_{i} } \right)$ is a parameter independent of time, the Laplace inversion of the above equation gives the expression for the collision force with respect to time. In collision theory, the notation $D\left( {A_{{\text{i}}} ,R_{i} ,l_{i} } \right)$ signifies that collisions happen instantaneously and are, therefore, not time-dependent; instead, they function solely as parameters. Specifically, A_instantaneous_, R_innstantaneous_, and L_instantaneous_ represent the instantaneous contact area, the instantaneous curvature radius of the contact surface, and the instantaneous thickness of the elastic body, respectively. This notation clarifies that instantaneous collision contact is not a function of time. However, during the entire collision contact process, variations in contact area and root depth are both time-dependent.

16 $$ F\left( t \right) = D\left( {A_{i} ,R_{i} ,l_{i} } \right) \cdot \ell^{ - 1} \left( {\overline{E}(s)^{*} } \right) \cdot \overline{\delta }(t)^{\alpha } \cdot \overline{V}\left( t \right) $$

$\ell^{ - 1} \left( {\overline{E}(s)^{*} } \right)$ Obtained in Simulink by means of transfer functions. The contact between the surface of the rubber wheel and the stepped discontinuous surface during the inter-stage transition of the variable diameter internal drive can be approximated as the contact between the elastic curved plate and the tapered surface, thus:

17 $$ D\left( {A_{i} ,R_{i} ,l_{i} } \right) = \frac{2AR}{{\pi {\text{tan}}\theta l}} $$

18 $$ E^{*} = E $$

19 $$ \alpha = 2 $$

where: $\theta$ is the vertebral angle of the cone, here it is considered that the step 90° is the least transition angle, for which the study is conducted. $A$ is the contact area. $R$ is the radius of the curved plate (rubber wheel radius). $l$ is the thickness of the curved plate (thickness of the rubber wheel). Among these equations, formula (17) is derived from the collision force parameter table, which is based on common contact surface properties. Since the contact between the elastic wheel and the stepped surface closely resembles the contact between the elastic curved plate and the conical surface, the basic collision force formula of 2/(π tan θ) serves as the foundation. To better align with the actual motion conditions^23^, additional parameters, including the contact area, rubber wheel radius, and thickness, are incorporated, taking into account structural size and shape parameters.

#### Instantaneous friction factors in collision contact

**Figure 5:**
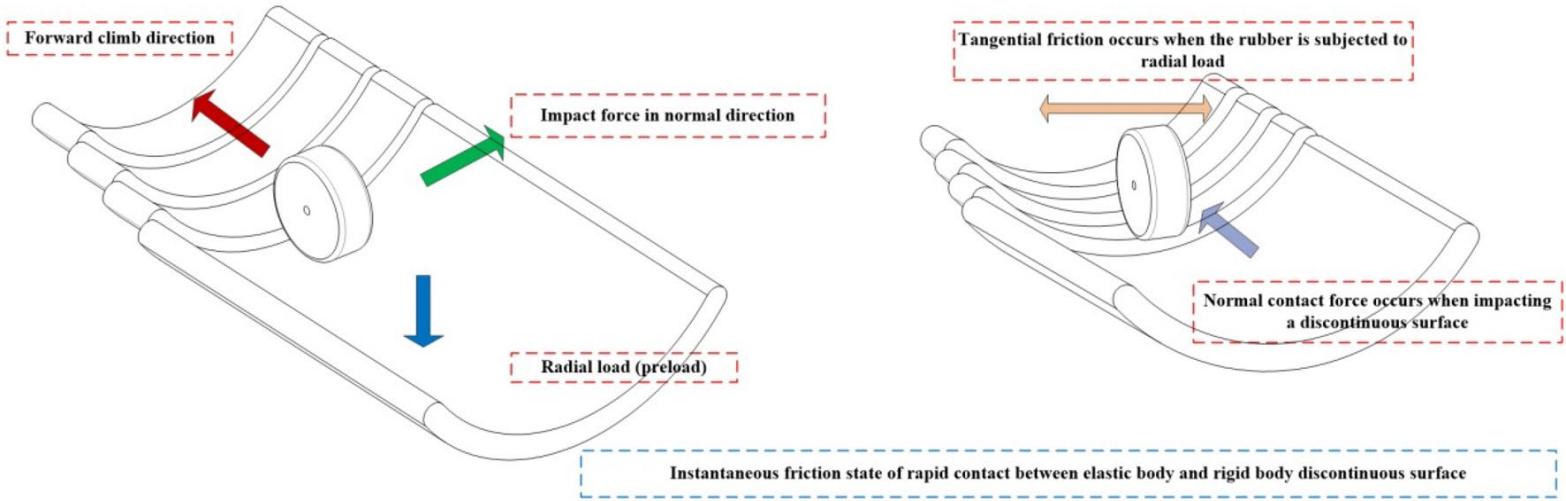
Schematic diagram of transient friction.

The model described above represents the collision force using a three-parameter solid model. However, it doesn't consider the normal contact load and tangential friction load between the two objects during transient contact within the contact area A. To address this, we develop an expression for intrusion between the two objects, taking into account the contact area, normal contact load, and tangential friction load. This expression is then integrated back into the collision force model, yielding the instantaneous friction state, as illustrated in Fig. 5.

Since the type of frictional contact at the time of collision is elastic rolling contact friction, the normal contact force^21^ at this time is:

20 $$ F_{{\text{N}}} \approx \frac{4}{3}E^{*} R^{1/2} d^{3/2} \approx \frac{16}{3}G_{\infty } R^{1/2} d^{3/2} $$

where $d$ is the depth of indentation, and $G_{\infty }$ is the modulus of elasticity, and $R$ is the radius of the rubber wheel. The tangential friction at this point contains rolling resistance and hysteresis friction caused by deformation.

For the resistance, there is: $F_{{\text{w}}} = F_{{\text{N}}} \frac{3}{4}\frac{v\tau }{R}$ where $F_{{\text{N}}}$ the normal contact forces, $v$ is the rolling speed, $\tau = \frac{{\overline{\eta }}}{{G_{\infty } }}$ is the relaxation time, and $R$ is the rubber wheel radius. The interaction between hysteresis friction and adhesive friction has been discussed in detail in^21^:

21 $$ F_{{\text{h}}} = K_{{\text{h}}} \frac{{E^{\prime\prime}}}{{E^{{{*}2}} p}}F_{{\text{N}}} ,F_{{\text{a}}} = K_{{\text{a}}} \frac{1}{{p^{r} }}E^{\prime\prime}F_{{\text{N}}} $$

where $K_{{\text{a}}}$ and $K_{{\text{h}}}$ are constants;$p$ is the normal force; the exponent $r = 0.2$; The $E^{\prime\prime}$ and $E^{*}$ are the rubber loss and the composite modulus, respectively. Therefore, the instantaneous tangential friction is:

22 $$ F_{{\text{q}}} = F_{{\text{N}}} \frac{3}{4}\frac{v\tau }{R} + K_{{\text{h}}} \frac{{E^{\prime\prime}}}{{E^{{{*}2}} p}}F_{{\text{N}}} $$

At this point the $F_{{\text{N}}}$ substitute into $F_{{\text{q}}}$ in the:

23 $$ F_{{\text{q}}} = \frac{16}{3}G_{\infty } R^{\frac{1}{2}} d^{\frac{3}{2}} \left( {\frac{3}{4}\frac{v\tau }{R} + K_{{\text{h}}} \frac{{E^{\prime\prime}}}{{E^{{{*}2}} p}}} \right) $$

At this time by the instantaneous tangential friction, the rubber volume deformation, volume deformation and the depth of indentation $d$ and contact area $A$ related to the volume deformation and the normal contact force of the relationship between.

24 $$ a^{3} = \frac{{3RF_{N} }}{{16G_{\infty } }} $$

At this point $a$ is the contact radius and the contact area is:

25 $$ A = 2a*l $$

At this time $l$ is the width of the rubber wheel.

Thus, after converting:

26 $$ \frac{A}{2l} = R^{1/2} d^{1/2} $$

At this time the $F_{{\text{q}}}$ contains the contact area $A$

27 $$ d^{\frac{3}{2}} = \left( {\frac{A}{{2l*R^{1/2} }}} \right)^{3} $$

The equations for the relationship between the normal contact force and tangential friction force and the contact area A in elastic collisions are obtained. Transformation of Eq. (23) into Eq. (17) follows:

28 $$ D\left( {A_{i} ,R_{i} ,l_{i} } \right) = \frac{{48l*R^{1/2} F_{{\text{q}}} E^{{{*}2}} pR^{2} \pi {\text{tan}}\theta l}}{{16\left( {3v\tau E^{{{*}2}} p + 4RK_{{\text{h}}} E^{\prime\prime}} \right)G_{\infty } R^{\frac{1}{2}} }} $$

The equation for the collision force at this point contains the normal contact force and tangential friction force in the elastic rolling phase.

29 $$ F\left( t \right) = D\left( {A_{i} ,R_{i} ,l_{i} } \right) \cdot \ell^{ - 1} \left( {\overline{E}(s)^{*} } \right) \cdot \overline{\delta }(t)^{\alpha } \cdot \overline{V}\left( t \right) $$

At this point, the collision force model contains the expressions of contact area $A$ for normal contact force and tangential friction, $R$ is the radius of the curved plate (rubber wheel radius), $l$ is the thickness of the curved plate (thickness of the rubber wheel), the $\delta$ is intrusion displacement of the two collisions, the $E^{*}$ is equivalent modulus of elasticity, and ${\text{V}}$ is collision velocity.

### Impact load model

**Figure 6:**
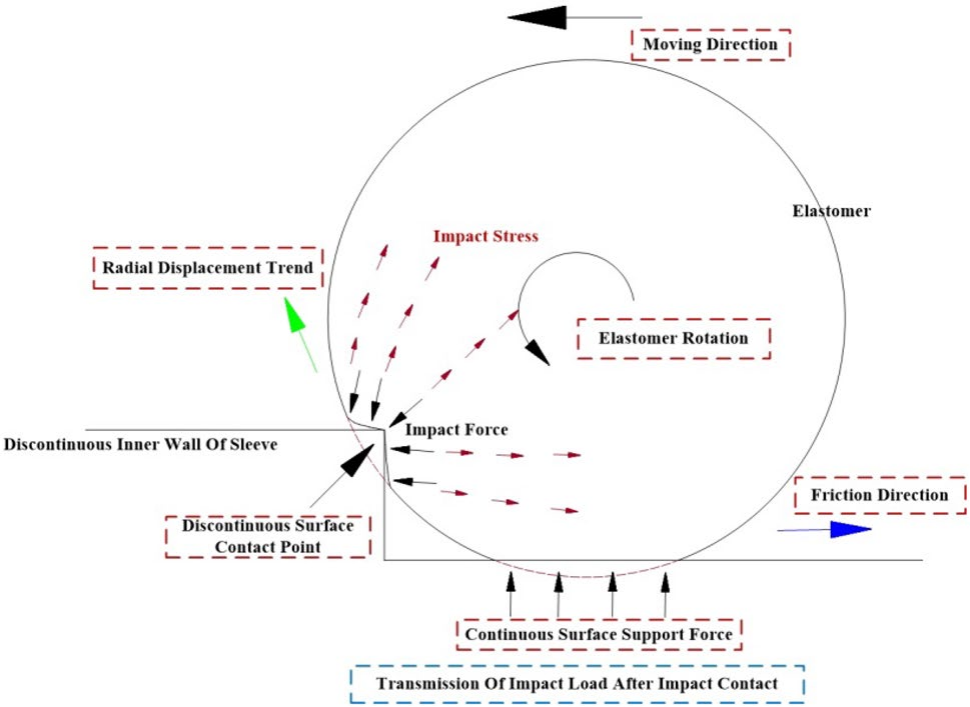
Schematic of impact load transfer at collision contact point.

Using the collision force as impact load, the stress transfer is always uniform in the stress distribution over the cross section inside the structure. Suppose considered as a microcell of length $dx$ of a microcell made of rubber, according to Newton's second law, there is:

30 $$ \rho Adx\frac{{\partial^{2} u}}{{\partial t^{2} }} = - \frac{\partial F}{{\partial x}}dx - \mu \frac{\partial u}{{\partial x}}dx + XAdx $$

where: $\rho$ Material density of rubber (kg/m^3^); $A$ Cross-sectional area of the microcell (m^2^);$u$ Axial displacement (m); $F$ the uniformly distributed force on the cross-section (N);$\mu$ the viscous damping coefficient when the micro-unit is rolling; $X$ the spatial position of a particle on a micro unit with a length of dx is represented by x. When observing the motion of matter in space, different particles arrive at this point in space at different times, that is, $X$ is a function of x and t, and X = X (x, t). The impact load transfer at the contact point is shown in Fig. 6.

The relationship between stress and strain by Hooke's law: $\sigma = - E{\mathcal{E}}$. Where $\sigma$ is the stress, positive in terms of compressive stress; ${\mathcal{E}}$ is the strain;$E$ is the modulus of elasticity of the rubber material.

31 $$ F = A\sigma = - AE\frac{\partial u}{{\partial x}} $$

The direction of $F$ is determined by its type: when it's a compressive force, the direction is positive, and when it's a tensile force, the direction is negative. This distinction arises because elastic materials undergo elastic deformation during collisions.

In mechanical systems, gravity can induce energy loss during the motion of objects, resembling a damping effect. This article specifically addresses the rubber wheel, constructed from elastic materials. However, given its lightweight and small volume, the impact force generated during mechanical work significantly surpasses its gravitational force. Furthermore, considering the device's operation in orbit within a zero-gravity environment, this damping effect will be temporarily excluded from consideration.

32 $$ \frac{{\partial^{2} u}}{{\partial t^{2} }} = c^{2} \frac{{\partial^{2} u}}{{\partial x^{2} }} $$

where $c = \sqrt {E/\rho }$, called the one-dimensional fluctuation equation, and $c$ is called the wave velocity, which is a physical quantity that responds to the properties of the material itself. A slight derivation using the above equation gives the system's set of control equations.

33 $$ \left\{ {\begin{array}{*{20}c} {\rho A\frac{\partial v}{{\partial t}} + \frac{\partial F}{{\partial x}} = 0} \\ {AE\frac{\partial v}{{\partial x}} + \frac{\partial F}{{\partial t}} = 0} \\ \end{array} } \right. $$

where $v$- the velocity of axial motion of the microcellular body $\left( {{\text{m}}/{\text{s}}} \right),$ $v = v\left( {x,t} \right) = \partial u\left( {x,t} \right)/\partial t$ .For the simplest case: the rubber wheel is of uniform material and texture; the analytical solution can be obtained in the form of D'Alembert.

34 $$ u\left( {x,t} \right) = \varphi_{1} \left( {x - ct} \right) + \varphi_{2} \left( {x + ct} \right) $$

This leads to:

35 $$ \left\{ \begin{gathered} v\left( {x,t} \right) = \frac{1}{Z}\left[ {P\left( {x - ct} \right) - Q\left( {x + ct} \right)} \right] \hfill \\ F\left( {x,t} \right) = P\left( {x - ct} \right) + Q\left( {x + ct} \right) \hfill \\ \end{gathered} \right. $$

Eq:$Z = \rho cA$,$Z$ is called the wave impedance ($N \, s/m$), characterizing the wave propagation capacity. Where:

36 $$ P\left( {x - ct} \right) = - AE\varphi_{1}^{\prime} \left( {x - ct} \right),Q\left( {x + ct} \right) = - AE\varphi_{2}^{\prime} \left( {x + ct} \right) $$

**Figure 7:**
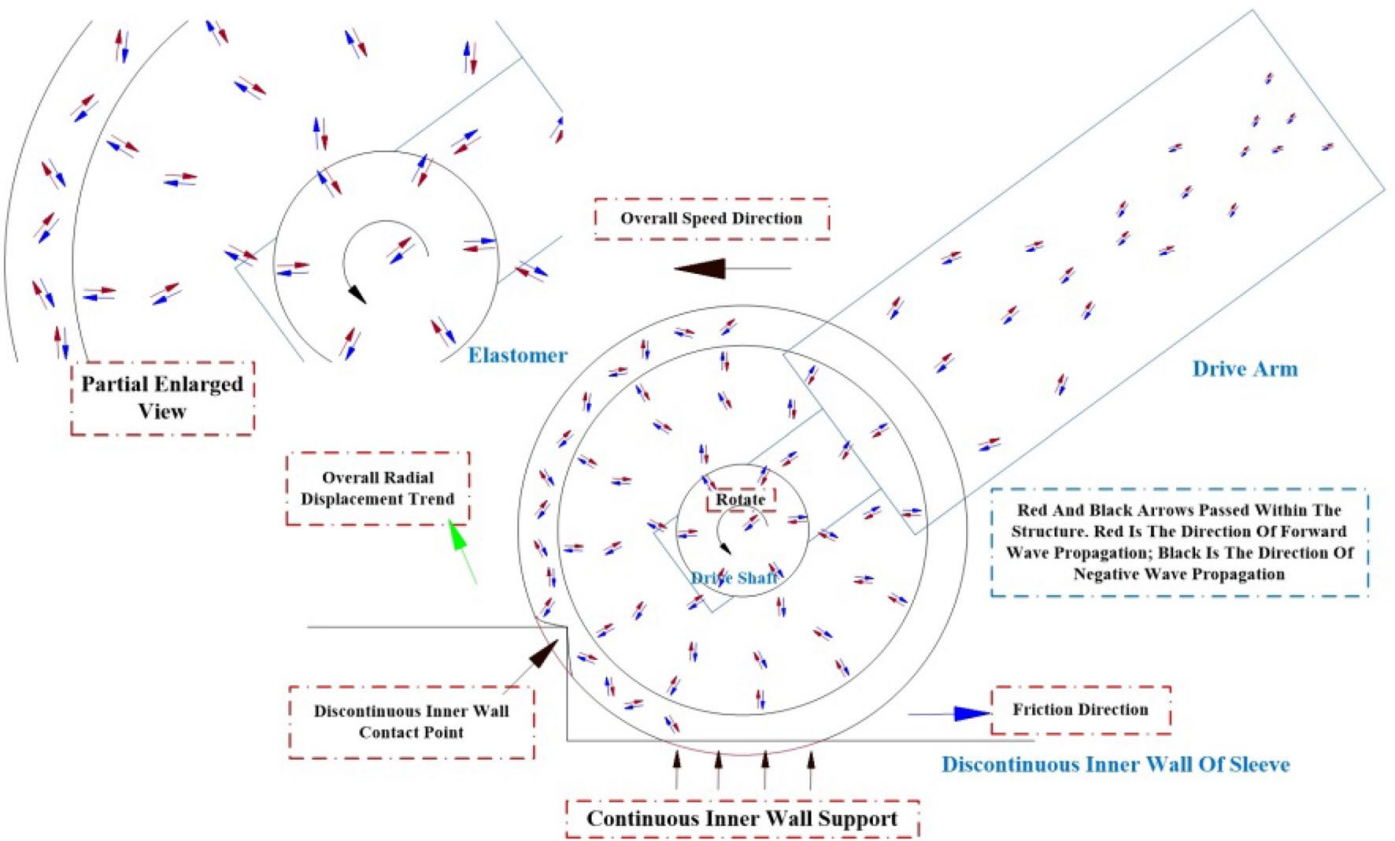
Transmission path of instantaneous positive and negative waves in structural collision.

$P$ and $Q$ respectively, denote the forces acting at wave speeds $c$.The two waves traveling in opposite directions are said to be traveling along the $x$ axis of the wave propagating in the forward direction $P$ is the positive wave, and the wave propagating along the $x$ axis $Q$ is the negative wave. The positive and negative wave transmission paths on the structure are shown in Fig. 7.

The viscoelastic material constitutive of the three-parameter collision model leads to the displacement $u$ equation as the unknown quantity, The stress changes ($\tau$) during the displacement process were derived from earlier investigations into elastic friction contact occurring in the interstage transition stage^21^:

37 $$ \frac{{\partial^{2} u}}{{\partial t^{2} }} - c^{2} \frac{{\partial^{2} u}}{{\partial x^{2} }} - c^{2} \tau \frac{{\partial^{2} u}}{{\partial x^{2} \partial t}} = 0 $$

where the equation:

38 $$ \sigma + p_{1} \dot{\sigma } = q_{0} \varepsilon + q_{1} \dot{\varepsilon } $$

The above equation is the intrinsic relationship equation of the three-parameter model. According to the equation with displacement $u$ equation as the unknown quantity, it is obtained that:

39 $$ u\left( {x,t} \right) = A{\text{exp}}\left( { - \alpha x} \right){\text{exp}}\left[ {i\left( {\omega t - kx} \right)} \right] $$

Among them:

40 $$ \alpha^{2} = \frac{{\rho E\omega^{2} }}{{2\left( {E^{2} + r^{2} \omega^{2} } \right)}}\left[ {\sqrt {1 + \frac{{r^{2} \omega^{2} }}{{E^{2} }}} - 1} \right] = \frac{{\omega^{2} }}{{2c^{2} \left( {1 + \omega^{2} \tau^{2} } \right)}}\left[ {\sqrt {1 + \omega^{2} \tau^{2} } - 1} \right] $$

Eq:$c^{2} = E/\rho ,\tau = r/E$ is the delay time,$k$ is a function of $\rho ,E,r,\tau .\omega$.

### Vibration transmission model

**Figure 8:**
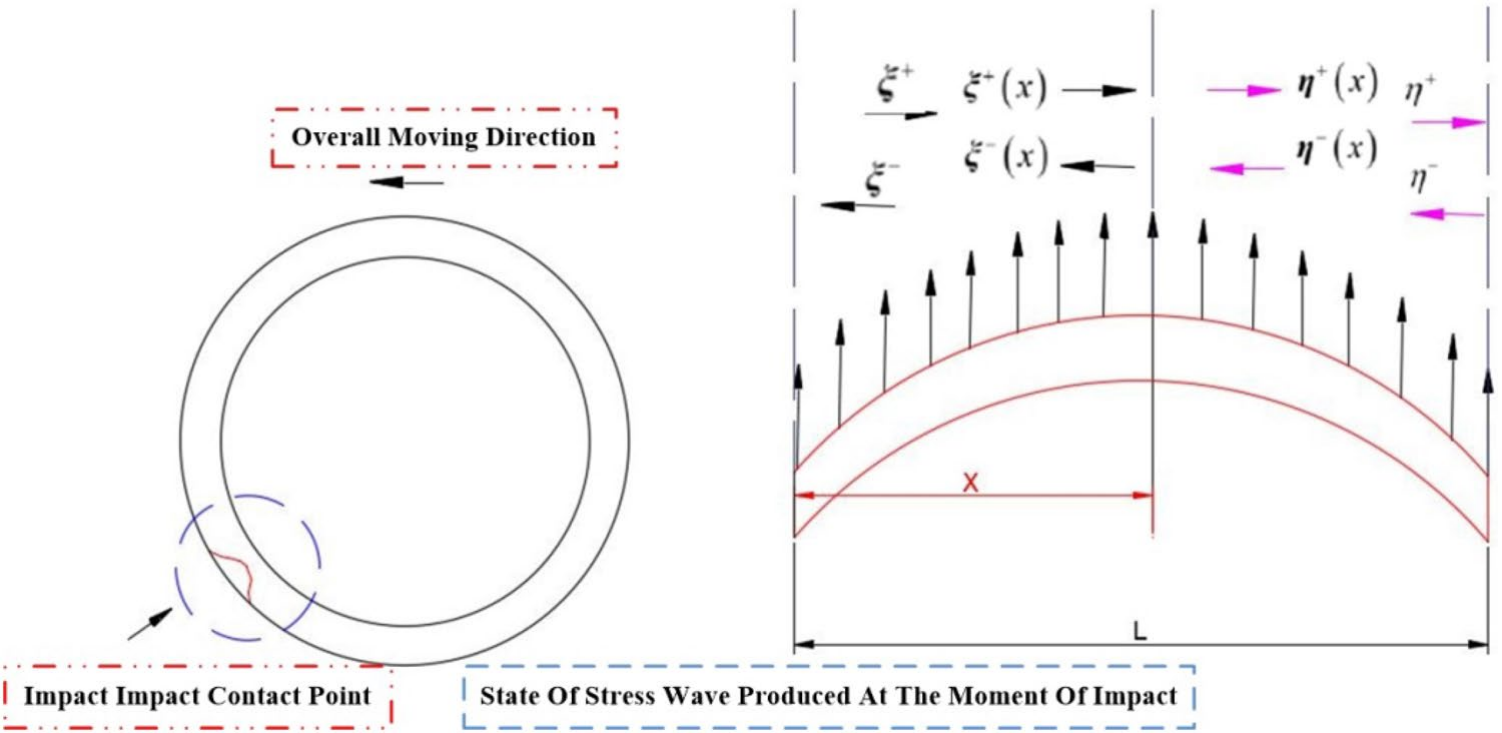
Distribution of stresses in viscoelastic structures under external excitation by stress waves.

From the impact load model of $\alpha$ term, the amplitude of the stress wave in the propagation process will be decayed as the propagation distance $x$ increases and decays continuously. Therefore, it is necessary to use the stress wave as the external excitation signal for the calculation of the vibration wave, as shown in Fig. 8, which is a schematic diagram of the distributed force of the viscoelastic structure under external excitation.

Since the wave vector method involves matrix operations, the amplitude response needs to be reduced to the oscillatory wave form using:

41 $$ W\left( x \right) = a^{ + } e^{ - ikx} + a^{ - } e^{ikx} + a_{N}^{ + } e^{ - kx} + a_{N}^{ - } e^{kx} = \left[ {\begin{array}{*{20}c} {1 \, 1} \\ \end{array} } \right]{\varvec{\eta}}^{ + } \left( x \right) + \left[ {\begin{array}{*{20}c} {1 \, 1} \\ \end{array} } \right]{\varvec{\eta}}^{ - } \left( x \right) $$

where $a^{ + }$,$a^{ - }$,$a_{N}^{ + }$ and $a_{N}^{ - }$ are the coefficients;$k$ is the wave number; the ${\varvec{\eta}}^{ + } \left( x \right)$ and ${\varvec{\eta}}^{ - } \left( x \right)$ are the forward and reverse waves of the stress wave, respectively.

42 $$ k = \sqrt {\frac{{\omega^{2} \rho \left( x \right)A\left( x \right)}}{E\left( x \right)I\left( x \right)}} ,{\varvec{\eta}}^{ + } \left( x \right) = \left[ {\begin{array}{*{20}c} {a^{ + } e^{ - ikx} } \\ {a_{N}^{ + } e^{ - kx} } \\ \end{array} } \right],{\varvec{\eta}}^{ - } \left( x \right) = \left[ {\begin{array}{*{20}c} {a^{ - } e^{ikx} } \\ {a_{N}^{ - } e^{kx} } \\ \end{array} } \right] $$

**Figure 9:**
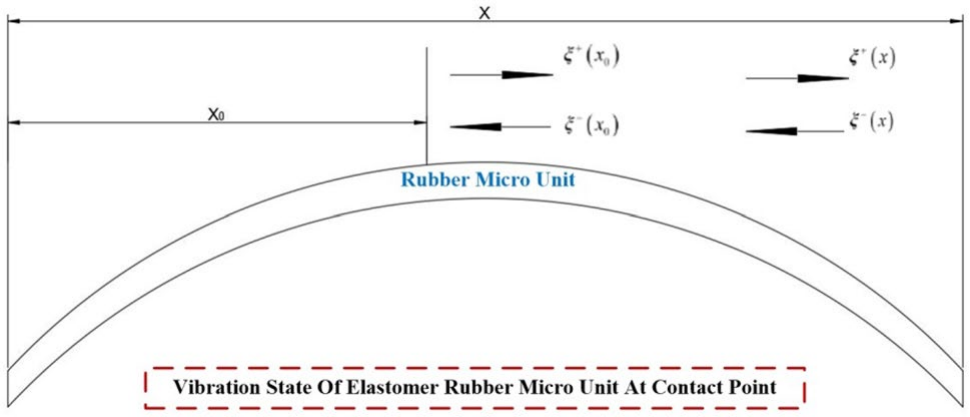
Vibration wave transmission diagram in the structure.

$x_{0}$ and $x$ there are positive and negative waves at the location, and the positive and negative vibration waves between the two points are shown in Fig. 9 with the following relationship:

43 $$ \left\{ \begin{gathered} \xi^{ + } \left( x \right) = \left[ {\begin{array}{*{20}c} {a^{ + } e^{ - ikx} } \\ {a_{N}^{ + } e^{ - kx} } \\ \end{array} } \right] = f\left( {x - x_{0} } \right)\xi^{ + } \left( {x_{0} } \right) \hfill \\ \xi^{ - } \left( x \right) = \left[ {\begin{array}{*{20}c} {a^{ - } e^{ikx} } \\ {a_{N}^{ - } e^{kx} } \\ \end{array} } \right] = f\left( {x_{0} - x} \right){\varvec{\xi}}^{ - } \left( {x_{0} } \right) \hfill \\ \end{gathered} \right. $$

Where $f\left( x \right)$ is the propagation matrix.

44 $$ f\left( x \right) = \left[ {\begin{array}{*{20}c} {e^{ - ikx} } & 0 \\ 0 & {e^{ - kx} } \\ \end{array} } \right] $$

From vibration wave transfer theory, if the positive and negative $x_{0}$ positive and negative waves at, then the amplitude vibration response at any point on the structure can be calculated by the following equation.

45 $$ W\left( x \right) = \left[ {\begin{array}{*{20}c} {1 \, 1} \\ \end{array} } \right]f\left( {x - x_{0} } \right){\varvec{\xi}}^{ + } \left( {x_{0} } \right) + \left[ {\begin{array}{*{20}c} {1 \, 1} \\ \end{array} } \right]f\left( {x_{0} - x} \right){\varvec{\xi}}^{ - } \left( {x_{0} } \right) $$

**Figure 10:**
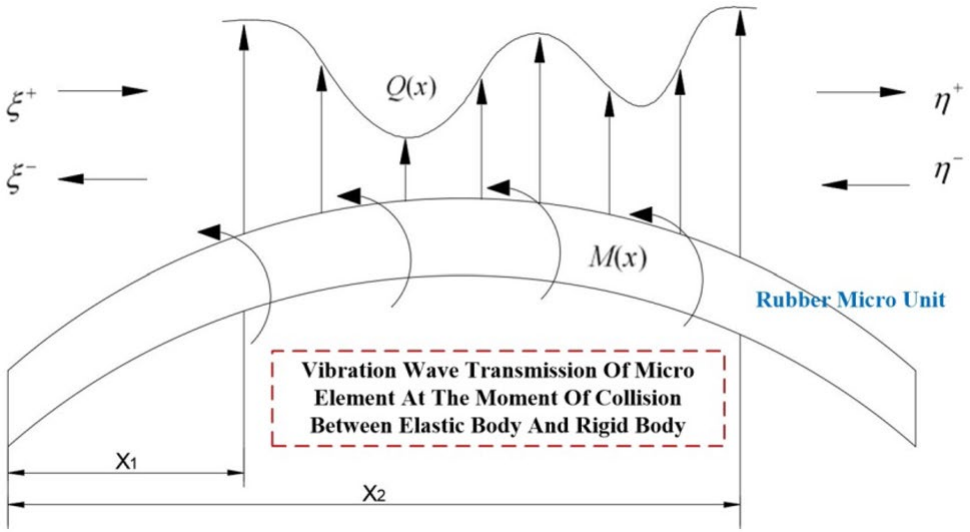
Transmission diagram of vibration waves at the moment of impact on the rubber wheel and the distribution of force and force couple.

During the aforementioned process, the transmission of vibration waves is often hindered by various constraints, with the discontinuous features exerting a significant influence on the wave transmission. In the case of a transient elastic collision, the rubber wheel experiences concentrated stress and bending moment. To address this, we can derive the equation by considering the continuity conditions of displacement and strain at the discontinuous features, as well as the equilibrium equation relating shear force and bending moment on both sides. This relationship is illustrated in Fig. 10.

46 $$ w_{1} = w_{2} ,\frac{{\partial w_{1} }}{\partial x} = \frac{{\partial w_{2} }}{\partial x},Q = F_{2} - F_{1} = EI\frac{{\partial w_{2}^{3} }}{{\partial x^{3} }} - EI\frac{{\partial w_{1}^{3} }}{{\partial x^{3} }} $$

47 $$ M = M_{1} - M_{2} = EI\frac{{\partial w_{1}^{2} }}{{\partial x^{2} }} - EI\frac{{\partial w_{2}^{2} }}{{\partial x^{2} }} \Rightarrow $$

48 $$ \left[ {\begin{array}{*{20}c} 1 & 1 \\ { - i} & { - 1} \\ \end{array} } \right]\left( {{\varvec{\eta}}^{ + } - {\varvec{\xi}}^{ + } } \right) - \left[ {\begin{array}{*{20}c} 1 & 1 \\ i & 1 \\ \end{array} } \right]\left( {{\varvec{\xi}}^{ - } - {\varvec{\eta}}^{ - } } \right) = 0 $$

49 $$ \left[ {\begin{array}{*{20}c} 1 & { - 1} \\ i & { - 1} \\ \end{array} } \right]\left( {{\varvec{\eta}}^{ + } - {\varvec{\xi}}^{ + } } \right) - \left[ {\begin{array}{*{20}c} 1 & { - 1} \\ { - i} & 1 \\ \end{array} } \right]\left( {{\varvec{\xi}}^{ - } - {\varvec{\eta}}^{ - } } \right) = \left[ {\begin{array}{*{20}c} {\frac{M}{{EIk^{2} }}} \\ {\frac{Q}{{EIk^{3} }}} \\ \end{array} } \right] $$

Then the following relationship is obtained:

50 $$ \eta^{ + } - \xi^{ + } = q + m,\xi^{ - } - \eta^{ - } = q - m $$

Among them:$q$ and $m$ are the dimensionless equalities and bending moment coefficients.

51 $$ q = \left[ {\begin{array}{*{20}c} { - i} \\ { - 1} \\ \end{array} } \right]\frac{Q}{{4EIk^{3} }},m = \left[ {\begin{array}{*{20}c} 1 \\ { - 1} \\ \end{array} } \right]\frac{M}{{4EIk^{2} }} $$

In order to further obtain the equation of the intrinsic frequency, first consider the problem of free vibration of the structure without discontinuous features, the reflection matrices at both ends are $R_{A}$ and $R_{B}$, respectively, and consider the case of free vibration of the structure if the vibration wave of the two ends in contact with each other with $\xi_{A}^{ + }$,and $\xi_{A}^{ - }$, and $\eta_{B}^{ + }$, and $\eta_{B}^{ - }$ are expressed, the relationship between the vibration waves is:

52 $$ \eta_{B}^{ + } = f\xi_{A}^{ + } ,\xi_{A}^{ - } = f\eta_{B}^{ - } ,\xi_{A}^{ + } = R_{A} \xi_{A}^{ - } ,\eta_{B}^{ - } = R_{B} \eta_{B}^{ + } $$

By the above equation, it is obtained that:

53 $$ \left[ {R_{A} fR_{B} f - I} \right]\xi_{A}^{ + } = 0 $$

where $I$ is the unit matrix, and $f = f\left( L \right)$ . For the elastomer, $\xi_{A}^{ + }$ the condition that there are non-zero real solutions leads to the condition that the determinant of the coefficient matrix should be zero. This equation is the frequency equation as follows.

54 $$ \left| {R_{A} fR_{B} f - I} \right| = 0 $$

By using equation $\left| {R_{A} fR_{B} f - I} \right| = 0$ can be calculated to find the intrinsic frequencies of each order, and then the modal shape at the corresponding frequency needs to be found. The modal shape calculation method based on the wave vector method is used. Based on Eq.$\left[ {R_{A} fR_{B} f - I} \right]\xi_{A}^{ + } = 0$ normalized $\xi_{A}^{ + }$ to obtain the normalized forward wave $\overline{\xi }_{A}^{ + }$ and then the normalized negative waveform is obtained from Eq.$\eta_{B}^{ + } = f\xi_{A}^{ + } ,\xi_{A}^{ - } = f\eta_{B}^{ - } ,$ $\xi_{A}^{ + } = R_{A} \xi_{A}^{ - } ,\eta_{B}^{ - } = R_{B} \eta_{B}^{ + }$ to obtain the corresponding normalized negative wave $\overline{\xi }_{A}^{ - }$ then the amplitude vibration response at any point on the structure, taking into account the discontinuous characteristics and free vibration, is:

55 $$ \overline{W}\left( x \right) = \left[ {\begin{array}{*{20}c} {1 \, 1} \\ \end{array} } \right]{\varvec{f}}\left( x \right)\overline{\xi }_{A}^{ + } + \left[ {\begin{array}{*{20}c} {1 \, 1} \\ \end{array} } \right]{\varvec{f}}\left( x \right)\overline{\xi }_{A}^{ - } $$

The amplitude value of any point on the structure is further obtained by the time domain response of each point on the structure, and the time domain response equation is:

56 $$ w\left( {x,t} \right) = \overline{W}\left( x \right)e^{j\omega t} $$

Finally by $w\left( {x,t} \right)$ the vibration amplitude of the rubber wheel when it is in contact with a non-continuous surface can be obtained.

**Figure 11:**
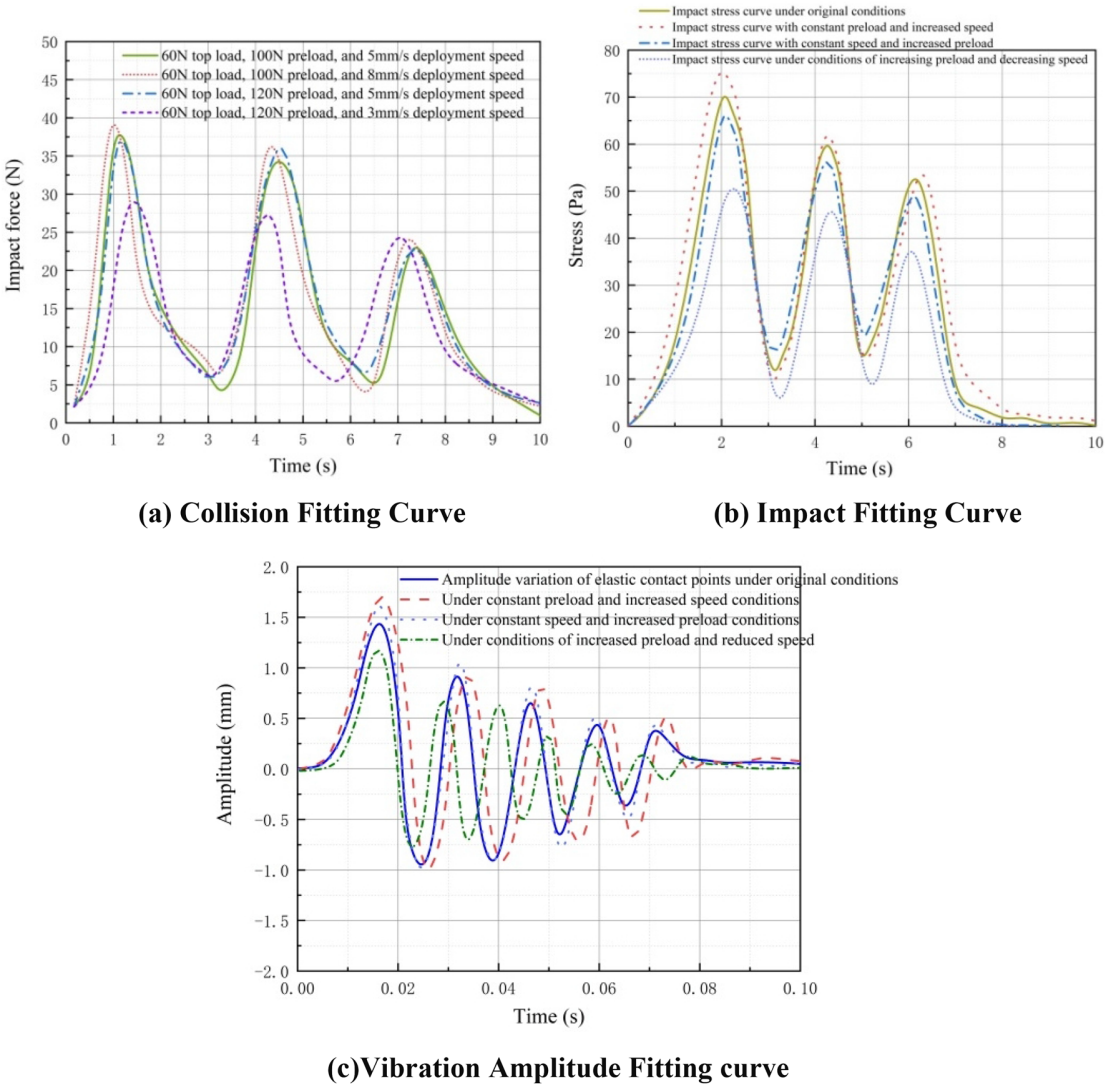
Fitting curves for collision, impact, and vibration under four parameter variable combinations.

To validate the suitability of the collision model, impact model, and vibration model, we examined four combinations of parameter variables: (1) load 60N, motion speed 5 mm/s, preload 100N; (2) load 60N, motion speed 5 mm/s, preload 120N; (3) load 60N, motion speed 8 mm/s, preload 100N; (4) load 60N, motion speed 3 mm/s, preload 120N. Figure 11 displays the fitting curves for collision, impact, and vibration corresponding to each of these four parameter combinations.

Figure 11a illustrates the contact between the elastomer and the rigid body. Due to elasticity, a periodic decay of the collision force occurs between the two. At a constant movement speed of 5 mm/s, a higher preload force results in a smaller collision force. Similarly, at a constant preload force of 100N, a higher movement speed leads to a greater collision-induced stress. Increasing the preload force to 120N yields consistent results. When the elastomer is subjected to different speeds and radial preload while in contact with the rigid body, the collision force generated on the elastomer is depicted in Fig. 11b. The impact load is transmitted through positive and negative waves, gradually decaying until reaching stability at zero. Figure 11c presents the fitting curves for vibration amplitude under different combinations of parameter variables. The curves demonstrate that for the same load and preload force, the vibration amplitude increases with higher speed, while for the same load and speed, a greater preload force results in a smaller change in vibration amplitude.

It is noteworthy that the impact response is influenced by the preload size. It has been observed that a larger preload leads to a smaller impact force. This phenomenon is applicable to the first stage of the sleeve in variable-diameter internal drive devices, where the diameter remains the same. As depicted in Fig. 11a, under the same load (60N), during the second peak stage within the second stage sleeve, a larger radial preload corresponds to a greater impact force. This occurs because, at the same speed, the sleeve diameter decreases, while the radial preload remains constant (100N and 120N in the first stage sleeve, and 100N and 120N in the second stage sleeve). This results in an increased compression rate of elastic materials, leading to a higher compression energy per unit area and consequently a greater impact force on their reaction. In the third stage, as the sleeve diameter decreases, a 20N difference in radial preload force has no significant effect on the compression rate of the elastic material. Hence, during the third peak, their impact forces are nearly identical.

In summary, the magnitude of the vibration amplitude is influenced by the vibration wave, which is generated by the stress wave. The stress wave, in turn, is determined by the difference between the collision force and the preload force. Therefore, adjusting the increase in preload force from the original output value effectively reduces the amplitude. This, in turn, leads to a more stable contact surface between the rubber wheel and the discontinuous surface during contact, with minimal changes to the contact area.

## Finite element analysis of elastic body during inter-stage transition

To verify the correctness of the contact state between the elastic body and the rigid body obtained from the elastic collision impact vibration theory in Section "Method" and whether changing the preload and motion speed effectively reduces the vibration amplitude, thereby improving the stability during contact, this section analyzes the motion state of elastic and rigid bodies using the finite element method. The analysis utilizes the static analysis module, Ls-dyna module, and vibration analysis module in Ansys Workbench and simulates them through coupled field analysis mode.

### Boundary conditions

At present, due to the presence of temperature control systems in space telescopes, it is possible to utilize certain temperature-limited materials in the aerospace field. The materials chosen for the simulation phase are generally similar to those used in ground-based variable-diameter preloading devices. The structural components are constructed from 2A12, known for its high tensile and yield strength, as well as excellent cutting performance. The pin shaft is composed of 40Cr, offering wear resistance and high load-bearing capacity. The gear is made from ultra-high-strength military-grade low alloy steel (45CrNiMoV). The spring selected is 60Si2MnA, demonstrating excellent mechanical properties after heat treatment. The drive wheel is crafted from a wear-resistant rubber material that exhibits stability under various conditions and a degree of impact resistance. In this study, butadiene styrene rubber has been chosen as the rubber material, which offers distinct advantages compared to other synthetic rubbers. In particular, the addition of white carbon black for vulcanization enhances its overall performance. The key performance parameters are listed in Table 1.

**Table 1:** Performance of butadiene rubber with added carbon black.

Function	2: 1. White carbon black vulcanizate	Function	2: 1. White carbon black vulcanizate
Density,${\text{kg}}/{{\text{m}}}^{3}$	1150	Thermal conductance $\text{W/ }({\text{m}}\cdot {\text{K}})$	0.3
Volume expansivity $\beta$	$530\times {10}^{-6}$	Dielectric constant (1kIlz)	2.5
Glass transition temperature K	221	Tensile strength MPa	17–28
Specific heat capacityKJ//kg⋅K	1.50	Tear strength KN/m	40–60

Upon analyzing the contact between elastic wheels and discontinuous surfaces from the perspective of elastic contact, it was discovered that a complete stepped surface transition comprises six stages, each associated with changes in the contact state and friction type. Previous research^21^ has identified these stages as follows: the third stage represents the contact state under static friction and viscoelastic friction loading; the fourth stage corresponds to the contact state during sliding friction and viscoelastic friction loading; the fifth stage characterizes the contact state during rolling friction and viscoelastic friction loading; and the sixth stage designates the contact state during sliding friction and viscoelastic friction loading (continuous surface stage under varying radial preloads). The first and second stages encompass the initial state and the state of applying radial preload, with the contact state being generally uniform at this point.

**Figure 12:**
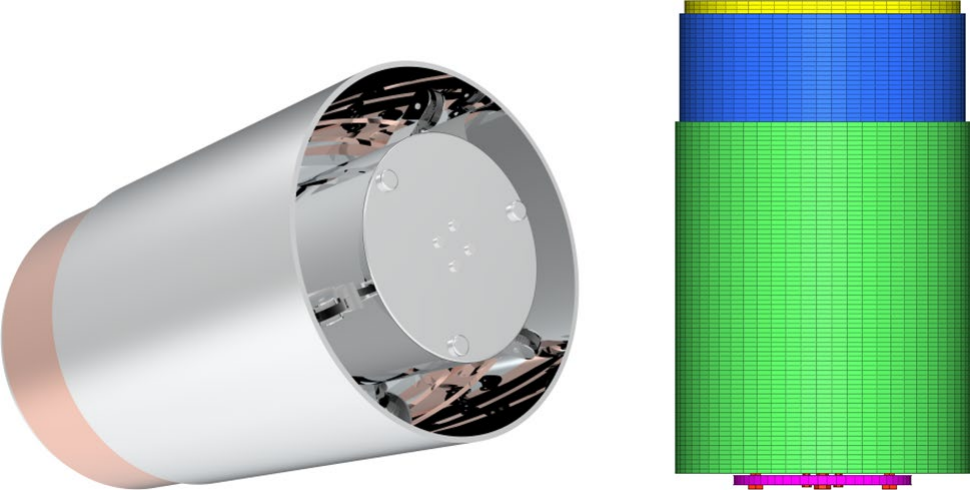
Structure of variable diameter internal drive device and finite element mesh model.

Additionally, the variable diameter internal drive device underwent further modeling and hexahedral meshing, as depicted in the Fig. 12. Given that variable diameter internal drive devices experience simultaneous tensile, compressive, and bending loads, the simulation employed beam elements, leading to a total of 7429 beam structures. Nonlinear elastic (nlstat) elements were used to simulate the flexible connection components, amounting to 1847 nonlinear elastic elements. Among these criteria, the minimum angle between grids should exceed 15 degrees, and the maximum angle should be less than 165 degrees. Additionally, the deformation value should be below 0.3, and the side length ratio should not exceed 2. One crucial indicator is the Jacques ratio, which ideally ranges from 0 to 1, with a higher ratio indicating better quality. In Fig. 12, the grid Jacques ratio is 0.92. During finite element analysis, especially for complex systems, the grid Jacques ratio should not fall below 0.85. Consequently, the grid quality depicted in Fig. 12 fulfills the prerequisites for finite element analysis.

Before commencing the analysis of collision, impact, and vibration, we established the initial external load conditions with a top load of 60 N. Given that the full structure includes a top load of 120 N and the designed variable diameter internal drive device features an axisymmetric structure with a 120° angle, we employed a half scale structure for validation. For boundary contact, we employed surface-to-surface contact, incorporating mixed contact modes, such as sliding friction, rolling friction, and viscoelastic friction within the contact bar. The contact state remained consistent in the axial direction. We enabled large deformation, particularly bending deformation, in the deformation settings, taking into account that the elastomer material used is JSR, which exhibits some elastic deformation when in contact with a rigid body with a discontinuous surface. The simulation time step is 0.01 s, in order to make the results as accurate as possible. The ambient temperature was maintained at 26 °C.

### Simulation analysis results

**Figure 13:**
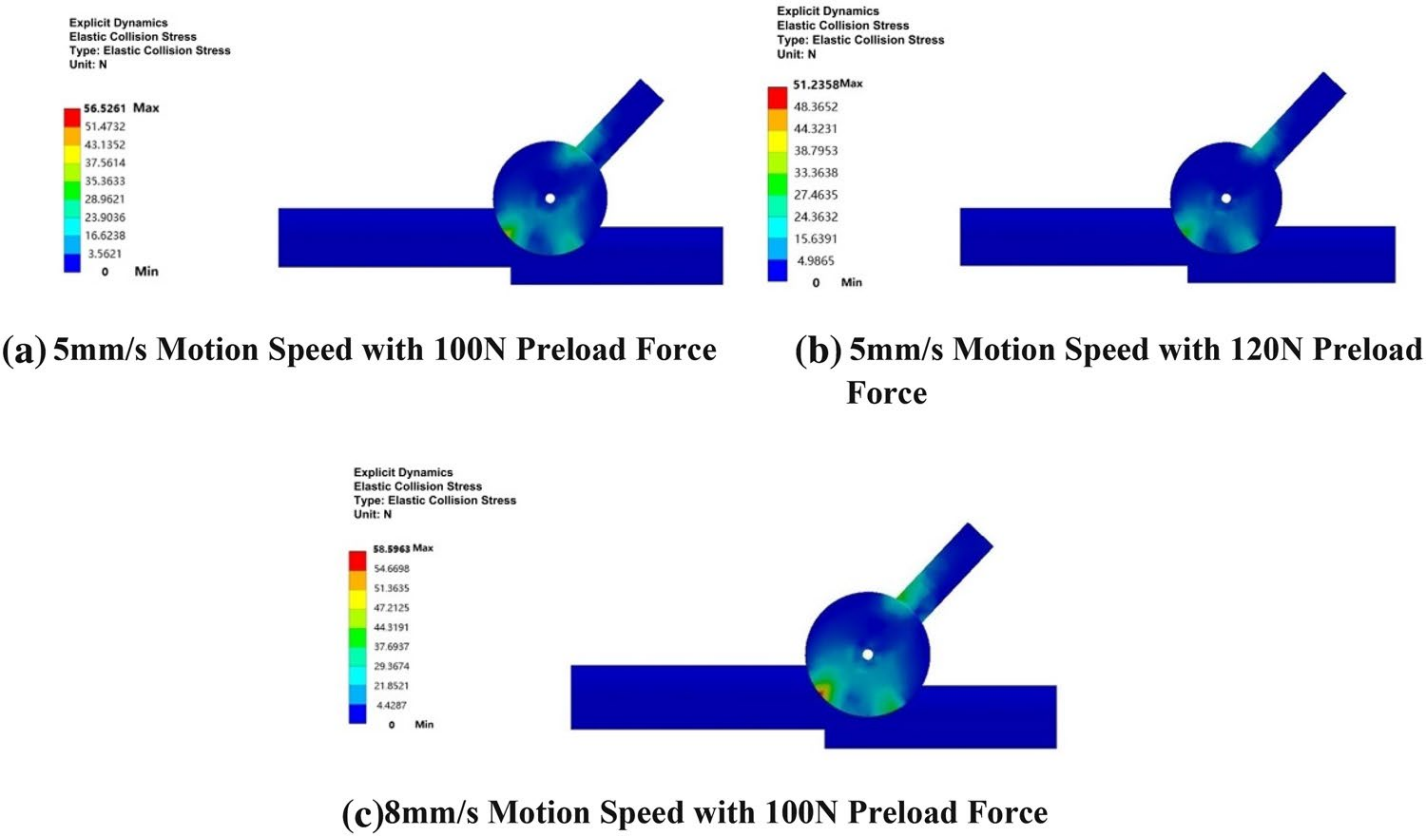
Inter-stage transition collision cloud chart.

To provide a clearer analysis of the contact state between an elastic body and a discontinuous rigid body surface, the simulation analysis diagram displays specific nonlinear stepped surfaces within the sleeve, as well as elastic rubber wheels, wheels, connecting pins, and certain driving arms. An initial axial load of 60N is applied, followed by separate collision analyses for the following combinations: 5 mm/s motion speed with 100N preload force, 5 mm/s motion speed with 120N preload force, and 8 mm/s motion speed with 100N preload force. The analysis results are shown in Fig. 13.

The collision cloud diagram for the inter-stage transition reveals intriguing insights. At a constant motion speed of 5 mm/s, an increase in preload force from 100 to 120 N leads to a reduction in elastic displacement during collision. This decrease in displacement is accompanied by a decrease in the elastic collision force, which drops from 56.5261 N to 51.2358 N, as illustrated in Fig. 13a,b. Conversely, maintaining a radial preload force of 100 N while increasing the motion speed from 5 mm/s to 8 mm/s results in an increase in elastic displacement during collision. The displacement rises from 56.5261 N to 58.5963 N, as demonstrated in Fig. 13a,c.

**Figure 14:**
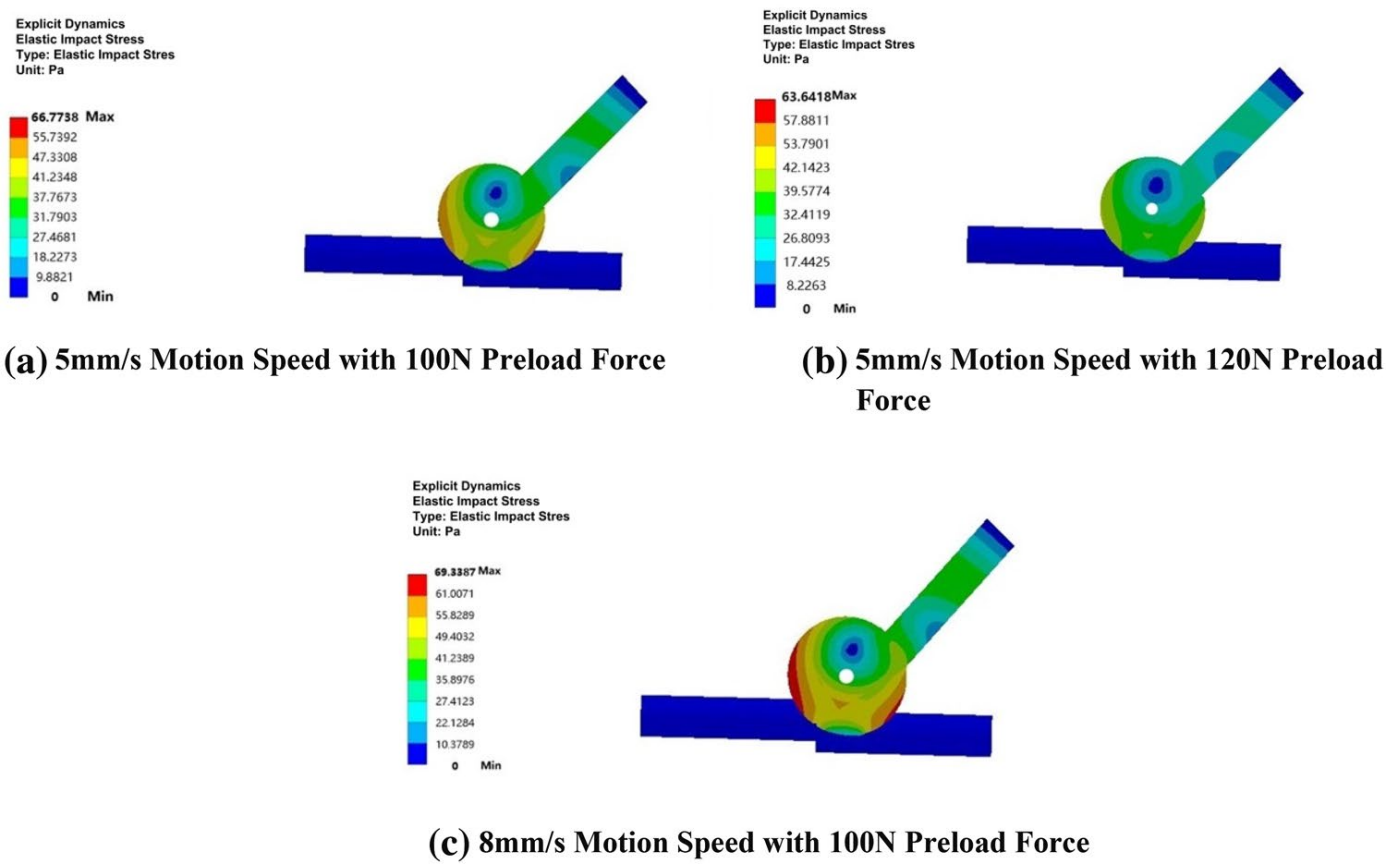
Inter-stage transition impact stress cloud chart.

The collision analysis results serve as essential boundary conditions within the impact module, depicted in Fig. 14a,b. These figures provide insights into the impact stress on the elastomer and the discontinuous rigid body step for a velocity of 5 mm/s and preload forces of 100 N and 120 N, revealing a decrease from 66.7738 Pa to 63.6418 Pa. Furthermore, Fig. 14a,c present stress clouds for a preload force of 100 N, highlighting the variations in impact stress with different motion speeds of 5 mm/s and 8 mm/s. In this scenario, the stress increased from 66.7738 Pa to 69.3387 Pa.

After a careful examination of the cloud diagram above, it becomes clear that, when the speed remains constant, the impact stress on the elastomeric rubber wheel decreases as the preload force increases. However, this analysis reveals a stress concentration on the drive arm, highlighting the need to enhance its strength to mitigate the concentration issue associated with higher preload forces. Conversely, when the radial preload force remains unchanged, the elastomer exerts higher impact stress at higher motion speeds. To further illustrate whether the stress generated during the elastic collision and impact process of the elastic body of the variable-diameter internal drive device, particularly in the first stage discontinuous rigid surface transition, aligns with the motion state, Table 2 displays the deformation displacement of the elastic body during elastic collision and impact simulations under different boundary conditions in the first stage inter-stage transition.

**Table 2:** Displacement during elastic collision and impact process.

Module	Operating mode	Stress	Displacement
Elastic collision	5 mm/s;100N	56.5261N	1.1661 mm
	5 mm/s;120N	51.2358N	1.1107 mm
	8 mm/s;100N	58.5963N	1.2717 mm
Elastic impact	5 mm/s;100N	66.7738pa	1.2811 mm
	5 mm/s;120N	63.6418pa	1.2410 mm
	8 mm/s;100N	69.3387pa	1.3439 mm

Theoretical studies suggest that as the impact stress intensifies, the strength of both positive and negative waves within the structural assembly also rises. Consequently, it is advisable to incorporate the impact stress results into the vibration analysis as a boundary condition.

**Figure 15:**
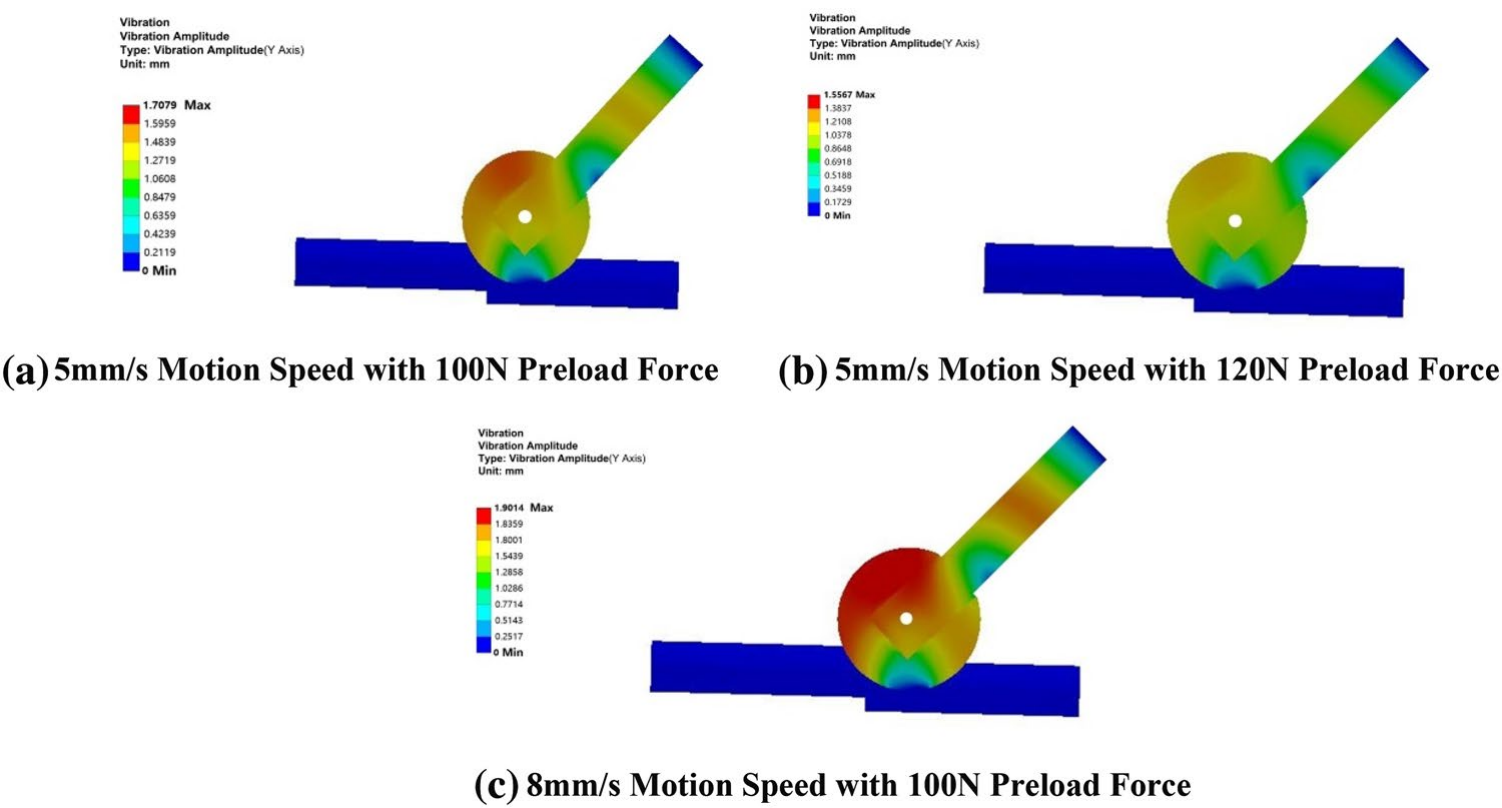
Inter-stage transition vibration amplitude cloud chart.

Figure 15 presents the variations in elastomer vibration amplitude under collision and impact conditions, specifically at a speed of 5 mm/s and with preload forces of 100 N and 120 N. In both panels (a) and (b), the amplitude displacement in the Y direction decreases from 1.7079 mm to 1.5567 mm. Similarly, when observing panels (a) and (c) with a constant preload force of 100 N but an increased speed from 5 mm/s to 8 mm/s, the cloud plot reveals an increase in amplitude displacement in the Y direction from 1.7079 mm to 1.9014 mm.

**Figure 16:**
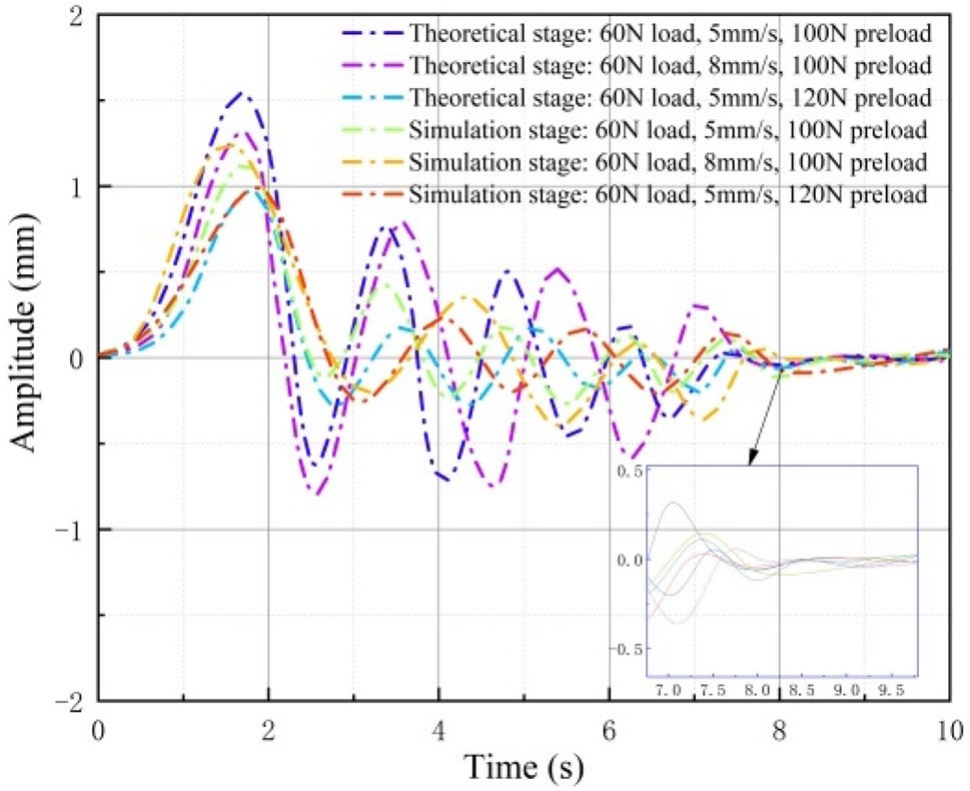
Vibration amplitude fitting curves for three different boundary conditions in theoretical and simulation stages.

An examination of the vibration amplitude cloud above indicates that the preload force effectively reduces the maximum vibration amplitude, while an increase in motion speed intensifies the vibration, resulting in a higher maximum vibration amplitude. To mitigate this instability in the contact state between the elastomer and the rigid body, it is advisable to increase the radial preload force during the stage transition. To visually and conclusively confirm the theoretical study's accuracy, we compare and analyze the vibration amplitude curves, as illustrated in Fig. 16.

It's important to note that, based on the theoretical analysis in Section "Method", the friction types at each stage of the inter-stage transition process are integrated into the elastic collision, while considering the constitutive model of the elastic body. This approach provides a more comprehensive consideration of changes in contact moments, considering both the material itself and external forces. Furthermore, it establishes theoretical connections between collision, impact, and vibration, aligning more closely with the actual motion state. To replicate the same motion state, we employ fundamental boundary conditions, including material properties and stress conditions. We incorporate the collision results as boundary conditions for the impact analysis through coupled field superposition, and subsequently, we employ the impact analysis results as boundary conditions for the vibration analysis module. Hence, as evident from the figure above, it is clear that the fitted vibration amplitude curves for three different boundary conditions during the simulation closely resemble those obtained from the theoretical study. Moreover, both the theoretical and simulation fitted curves indicate that higher preload forces result in smaller vibration amplitudes at the same speed, whereas higher speeds lead to larger vibration amplitudes at the same preload force. These findings underscore the influence of elastomer motion speed and radial preload on the contact state between the elastomer and the rigid body. They also emphasize the effectiveness of adjusting the radial preload to address variations in contact gap caused by speed.

## Experimental and discussion

### Experiment scheme

In order to verify the impact of changing motion speed and preload on the contact state between the elastic body and the rigid body during the transition stage of the variable diameter internal drive device, a 1:1 scale experimental prototype was designed and manufactured. Given the central axis-symmetric structure of the variable diameter inner drive device, comprising 6 identical variable diameter drive components, one of these components (equivalent to 1/6 of the variable diameter inner drive device) was selected as the experimental prototype for elastic collision contact testing and discussion during the stage transition. It's important to note that the size parameter ratio of the test prototype to the variable diameter drive component of the complete variable diameter internal drive device is 1:1. The choice of materials aligns with the theoretical and simulation phases. Adjusting the positions of the driving motor and pre-tightening motor necessitated the calculation of traction force and radial pre-tightening force on the wheels at the theoretical stage, recalibrating the output values to match the boundary conditions employed in the simulation stage.

**Figure 17:**
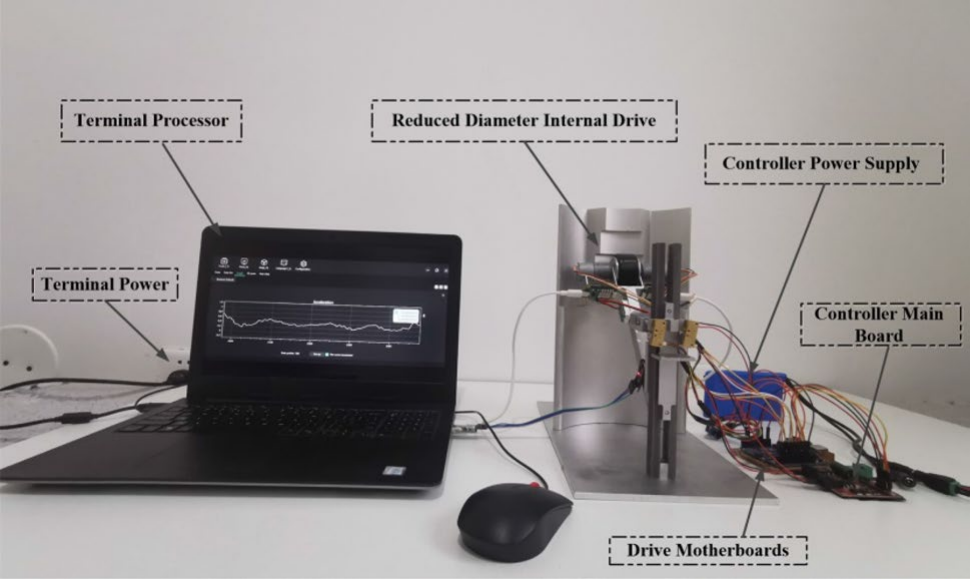
1:1 experimental prototype image.

As depicted in Fig. 17, the experimental prototype is primarily divided into a sleeve support section and a variable diameter drive pre-tightening section. The sleeve support section provides geometric constraints for the variable diameter drive pre-tightening section, allowing it to move axially along the inner wall surface of the sleeve. The sleeve support section mainly comprises a base, a localized structure of the sleeve, a rear support, and a central pillar. The distance between the central pillar and the localized structure of the sleeve with the maximum outer diameter is 150 mm. The height of the central pillar is 215 mm, and the localized structure of the sleeve is 280 mm high, with a spacing radius of 4 mm at each level. The variable diameter drive pre-tightening section supplies axial and radial pre-tightening force to the rubber wheel. This section primarily includes the rubber wheel, a driving arm, a sliding connector, a pre-tightening component, and a motor set. The rubber wheel has a radius of 25 mm and a width of 28 mm. The length of the driving arm is 145 mm, with a cross-section of 12 × 15 mm. The pre-tightening component can extend from 12 to 35 mm, and the sliding connector has an axial displacement of 85 mm.

**Figure 18:**
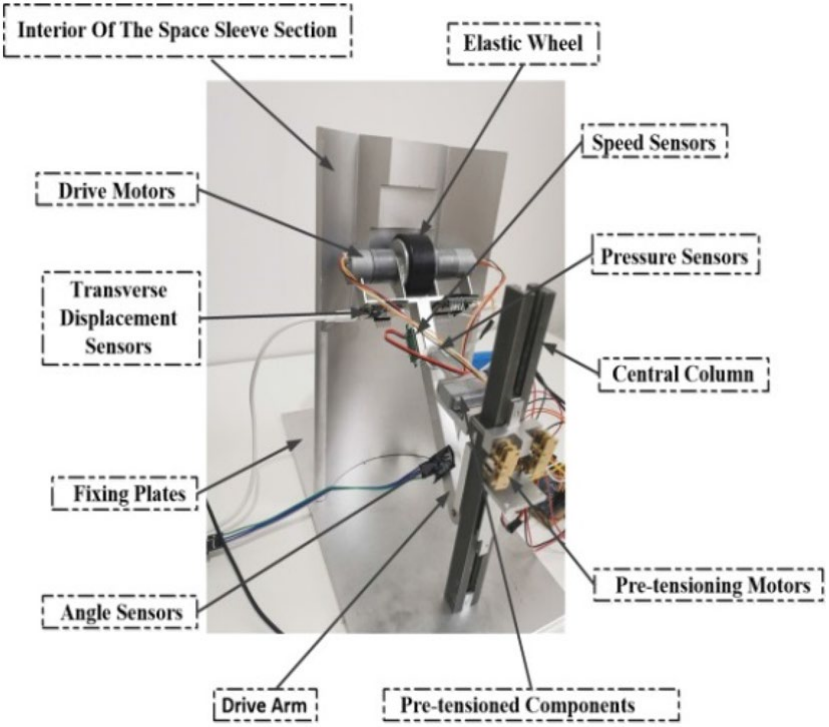
Sensor installation location schematic.

In the experiment, various sensors were employed to measure and monitor the behavior of the elastic body. These sensors included velocity sensors, pressure sensors, displacement sensors, and angle sensors. Figure 18 illustrates the specific installation positions of each sensor within the experimental prototype.

The velocity sensor was placed on the side of the drive arm to measure the rotational speed of the fixed point on the rubber wheel's side, allowing us to determine the movement speed. The pressure sensor was located at the connection point between the pre-tightening component and the driving arm, and it provided output values for the radial pre-tightening force by measuring the mutual force between the two. A displacement sensor was situated at the connection between the driving arm and the driving wheel, facilitating the measurement of changes in the rubber wheel's width after compression during movement, thanks to calibration. Meanwhile, an angle sensor was positioned at the connection point between the driving arm and the central pillar. It allowed for the measurement of angle changes during movement by calibrating the initial angle value between the two. This data was essential for determining whether the variable diameter internal drive device's movement was complete, thereby providing a signal for the motor's start-stop program.

These sensors collectively provided information about the rubber wheel's movement speed, radial preload load, compression amount of the rubber wheel (which could be converted into the contact area between materials), and the offset angle of the rubber wheel. To ensure the test's consistency with the actual scenario, a load of 60N was applied to the variable diameter inner drive device.

The purpose of utilizing these four types of sensors and their specific installation positions, as illustrated in the figure, was to acquire data on vibration amplitudes. This data helped us determine the contact state between the elastic body and the discontinuous rigid step surface when they made contact at different speeds and preloads.

**Figure 19:**
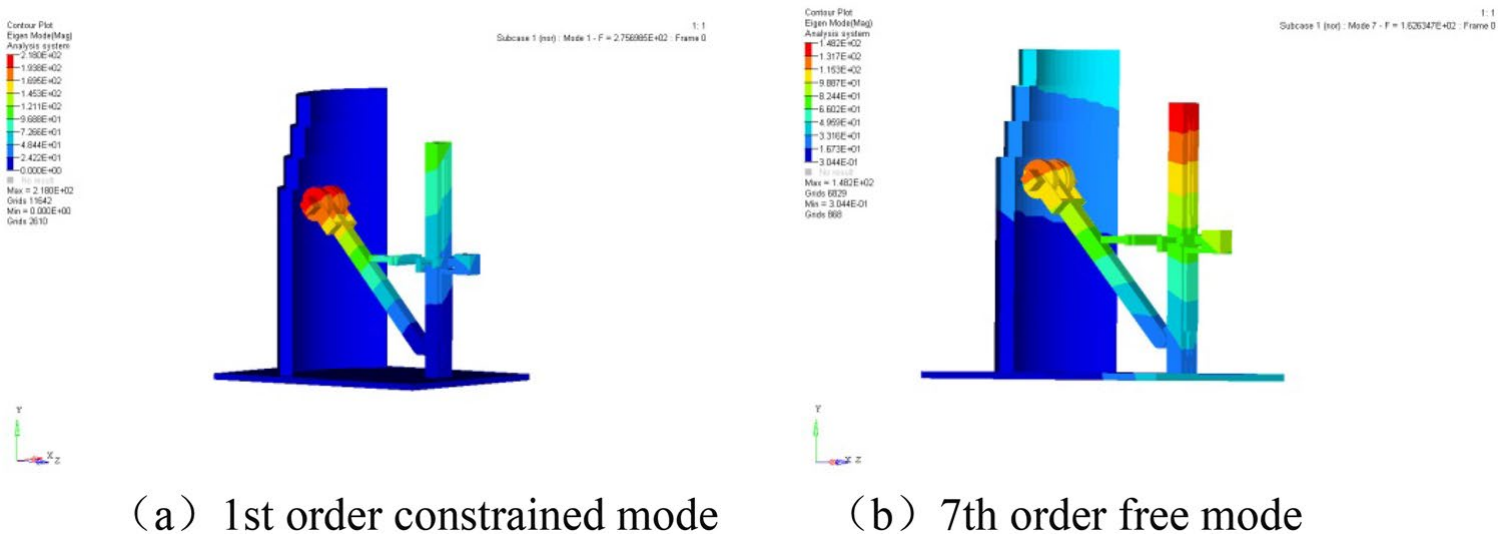
Constrained and free modes of the experimental setup.

It is noteworthy that modal analysis was specifically conducted on the experimental device to ensure that the design does not introduce coupling problems. This analysis provides a comprehensive understanding of the vibration behavior of the device across various frequency ranges, contributing to its improved performance and safety. Figure 19 illustrates the first constrained mode and the seventh free mode of the experimental device. From a practical engineering perspective, the constrained mode primarily observes the states of the first six orders, while the free mode focuses on the seventh order. In Fig. 19, the first constrained mode is observed at 276 Hz, and the seventh free mode at 163 Hz. The values of the first six orders of the constrained mode are detailed in Table 3.

**Table 3:** Values of the first 6 constrained modes of the experimental device.

Constrained modal order	Natural frequency value
1st order	276 Hz
2nd order	296 Hz
3rd order	503 Hz
4th order	674 Hz
5th order	749 Hz
6th order	827 Hz

### Analysis and discussion of test results

**Figure 20:**
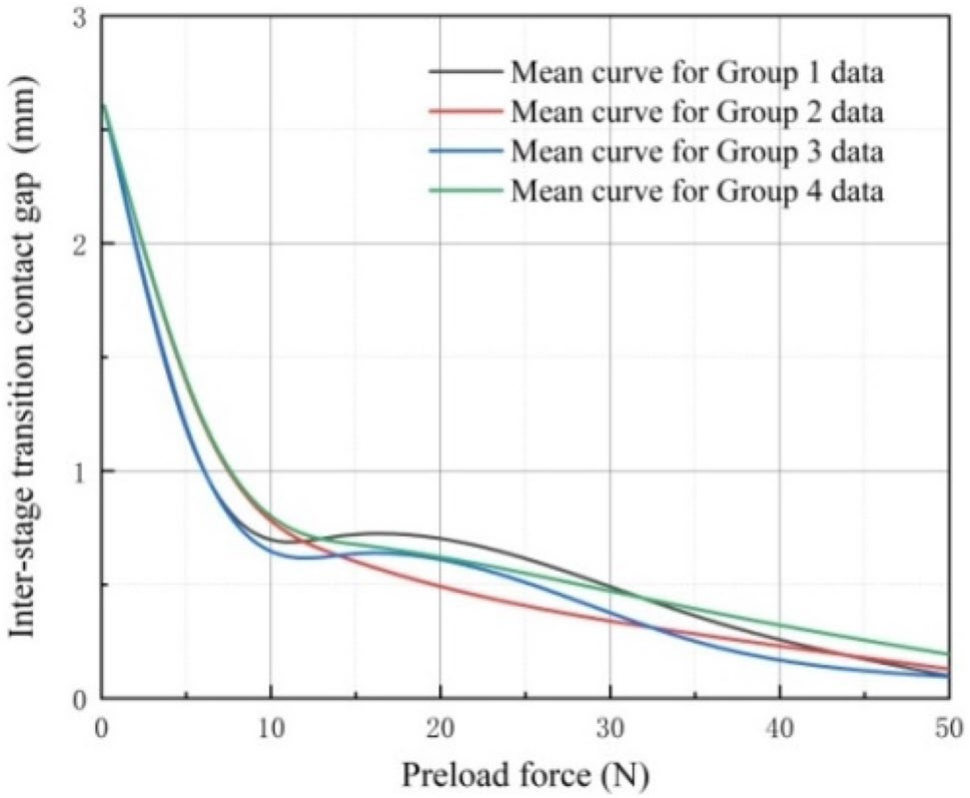
Preload force and contact gap data curves during inter-stage transition.

During the experimental process, the variable-diameter internal drive device underwent a complete three-stage transition and ascended to the top over a duration of 10 s. Utilizing a sensor to capture values, data was recorded every 0.1 s in the terminal processor. After completing 40 sets of experiments, each set of data underwent fitting, and 10 sets of data were randomly selected for averaging. Ultimately, four averaged curves were obtained, as illustrated in Fig. 20. The trends observed in the four mean curves depicted in the figure are consistent. Specifically, when the movement speed of the elastic wheel remains constant, an increase in preload during the transition between stages leads to a reduction in the contact gap between the elastic wheel and the discontinuous surface.

It's essential to note that using the same experimental approach, 40 sets of data were gathered. Due to the presence of minor discrepancies, the results of mean fitting exhibited slight variations for every 10 sets. Nevertheless, during the transition between stages, the consistent trend of changes in contact gaps due to their motion state and movement speed was observed. A deeper analysis was conducted to identify the sources of these errors, which were attributed to rubber wheel wear, delayed output electrical signals related to preload, and errors in part processing. However, during the transition between stages, these factors do not impede the observation of boundary conditions affecting changes in contact gaps. Among these four sets of mean variation curves, the maximum difference in contact gap is 0.24 mm.

**Figure 21:**
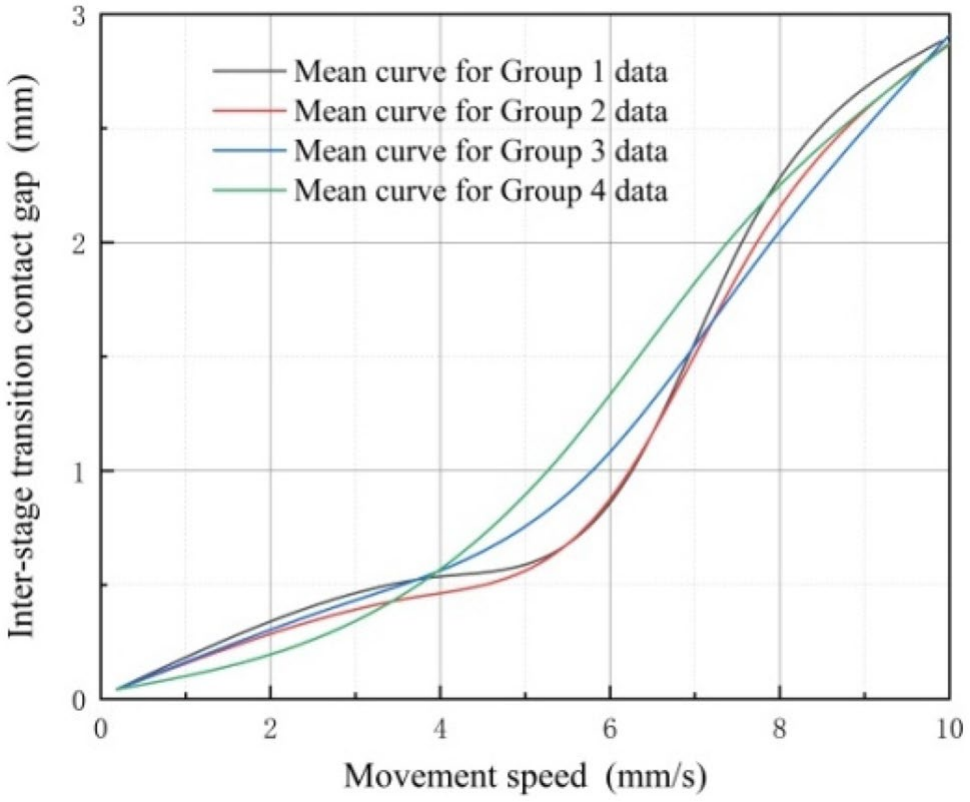
Motion speed and contact gap data curves during inter-stage transition.

Moreover, Fig. 21 illustrates that when the preload force supplied by the preload assembly remained consistent, the contact gap between the elastic wheel and the discontinuous surface exhibited a nonlinear expansion with the increase in movement speed. During this stage, a change occurs in the mean values of the four groups, primarily resulting from rubber wear influencing the contact and causing momentary elastic wheel slippage, even though this situation lasts for a very brief period. Among these four sets of mean curves, the maximum difference in contact gap is 0.32 mm.

**Figure 22:**
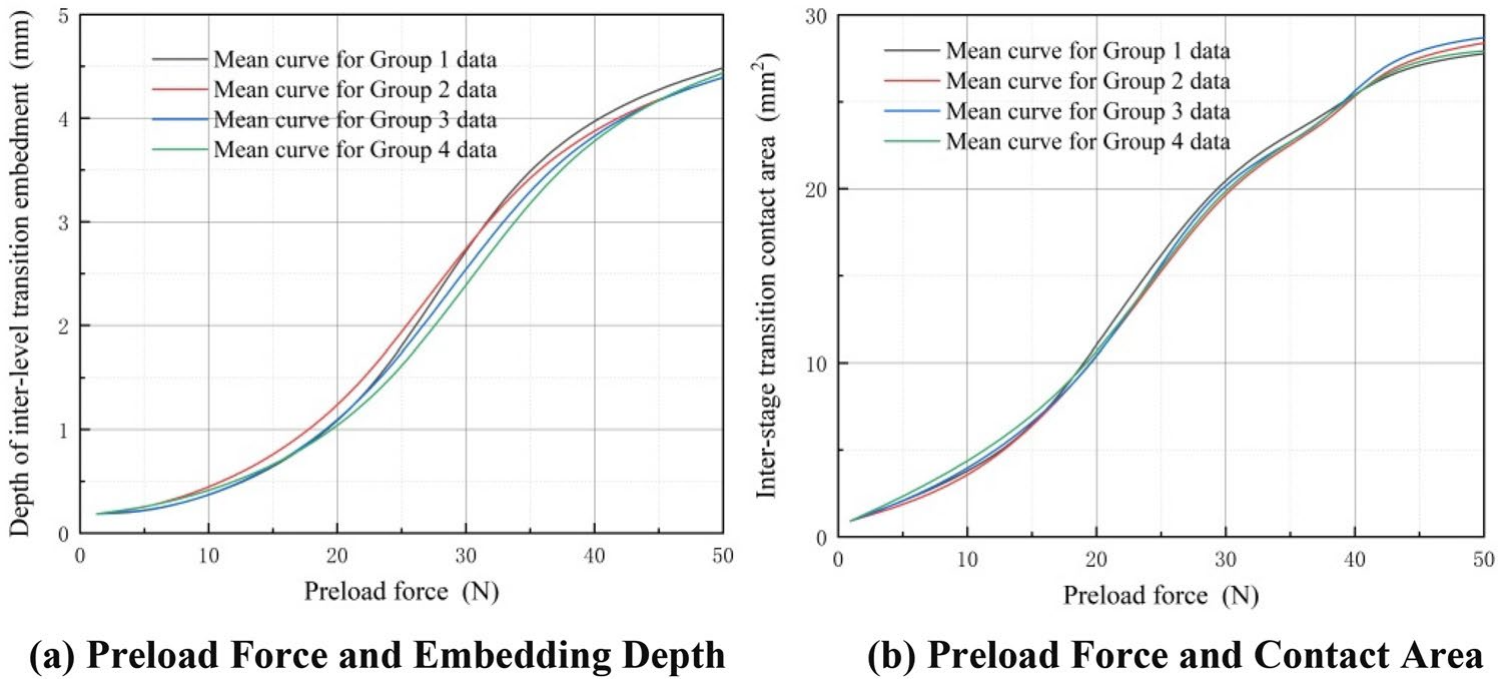
Inter-stage transition preload force, elastic wheel embedding depth, and contact area data curves (constant speed).

In the experiment, the normal contact distance of the displacement sensor is measured during the transition between stages to determine the embedding depth of the elastic body when it is embedded on the surface of a discontinuous rigid body. Displacement sensors are utilized to measure the contact length of the elastic body when it makes contact with the inner wall of the sleeve. Subsequently, another set of displacement sensors is employed to measure the lateral extrusion displacement of the elastic body. This process ultimately yields the contact area between the elastic body and the surface of the discontinuous rigid body. In Fig. 22a, it is evident that at a constant speed, the inter-stage transition between the elastic wheel and the discontinuous surface shows a nonlinear increase in the embedding depth as the preload force increases. Furthermore, an observation can be made that as the embedding depth increases, the contact area between the elastic wheel and the discontinuous surface also increases, as depicted in Fig. 22b. Similarly, the change in mean, as indicated in Fig. 18, is attributed to errors. In Fig. 22a, the maximum difference in mean changes among the four groups is 0.18 mm. Figure 22b, on the other hand, reveals a more significant disparity, with the maximum difference reaching 2.04mm^2^.

**Figure 23:**
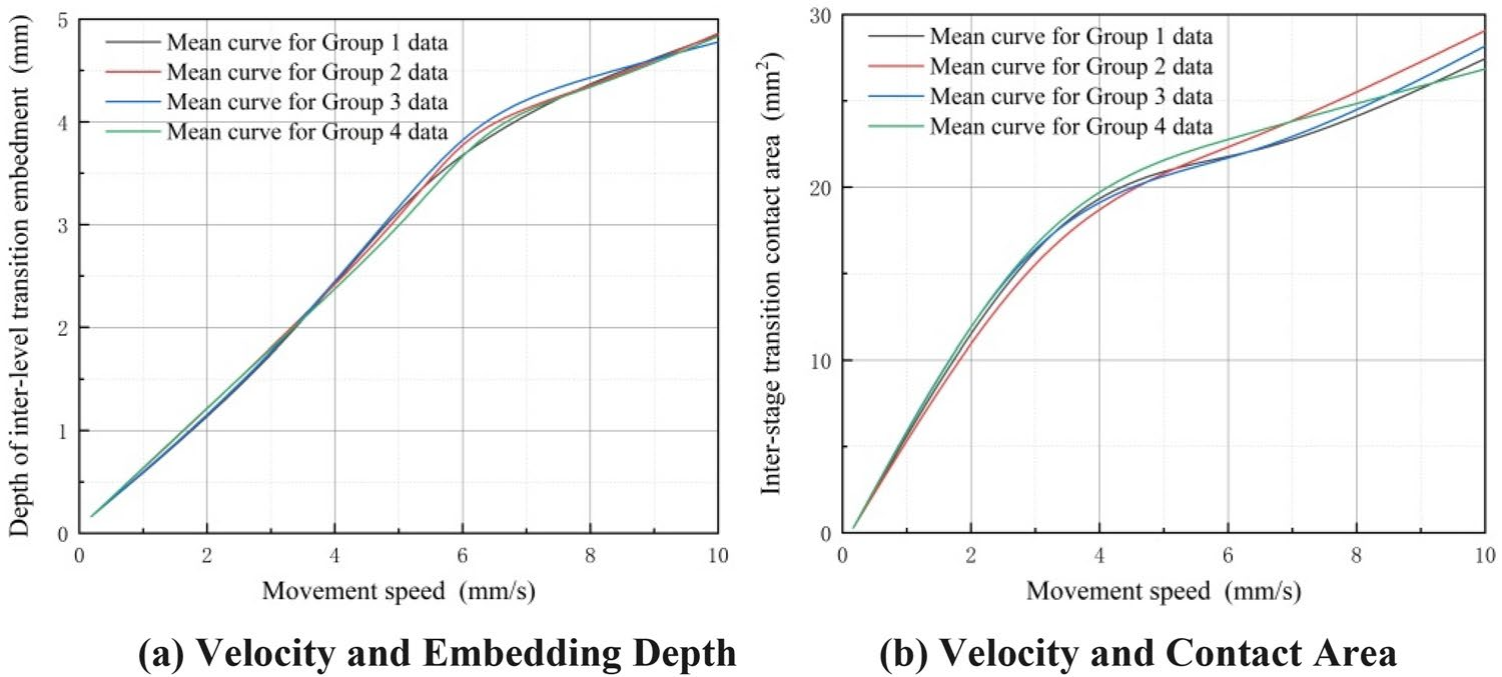
Inter-stage transition motion speed, elastic wheel embedding depth, and contact area data curves (constant preload force).

Figure 23a shows that, under a constant preload force, the inter-stage transition phase leads to an increase in the embedding depth of the elastic wheel as its moving speed rises. Furthermore, Fig. 23b illustrates that the contact area between the elastic wheel and the discontinuous surface gradually expands as the embedding depth grows. Likewise, Fig. 23a reveals that the maximum difference in mean changes among the four groups is 0.21 mm. In contrast, Fig. 23b shows a more substantial disparity, with the maximum difference reaching 2.71mm^2^.

To explore the contact state between the elastomer and the rigid body under non-low speed conditions, a controlled test was conducted. This test included variations in both speed and preload force, as detailed in Table 4, to monitor the changes in the contact state during the inter-stage transition.

**Table 4:** Experimental values during inter-stage transition phase.

Status	Stage	Gap changes(mm)	Depth of embedding(mm)	Contact area(mm^2^)
Constant speed	Transition phase 1	0.96	3.11	21.02
	Transition phase 2	0.91	3.06	20.86
	Transition phase 3	0.85	3.01	20.61
Constant preload force	Transition phase 1	1.42	4.36	22.32
	Transition phase 2	1.44	4.41	22.51
	Transition phase 3	1.48	4.45	22.64

**Figure 24:**
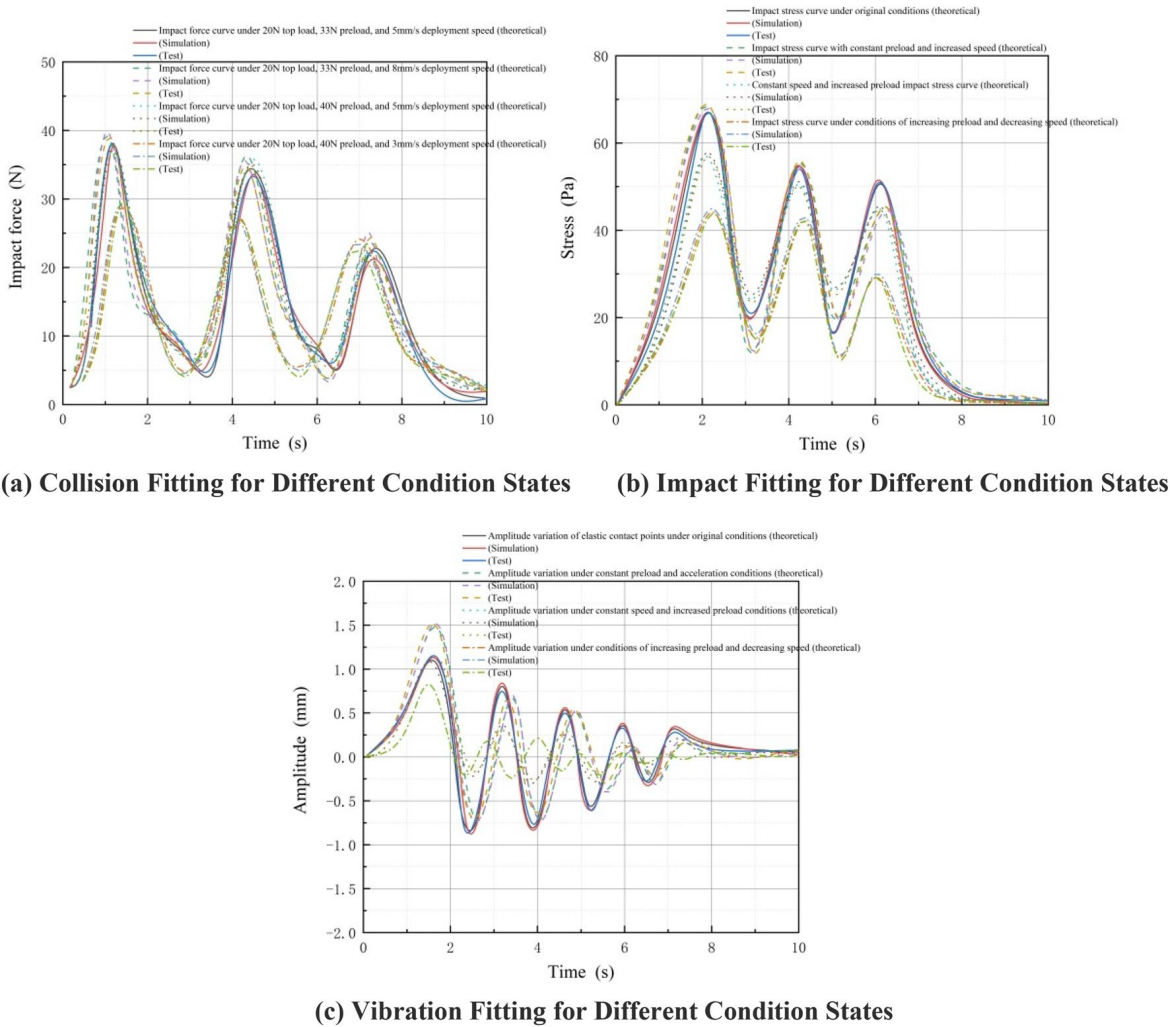
Fitting curves for collision, impact, and vibration under different condition states during the experimental stage of inter-stage transition.

The conducted tests revealed significant impacts of speed and preload force on the collision between the elastomer and the rigid body. Additionally, collision, impact, and vibration curves were obtained for various condition states, as illustrated in Fig. 24.It is important to note that, during the testing phase, a complete variable-diameter internal drive device was not available due to its unique structure. To address this limitation, one-third of the results were selected for testing to adequately explain and verify the research content of this article. Consequently, the legend in Fig. 22 indicates a top load of 20N and a radial preload of 33N-40N. In the test depicted in Fig. 22, the maximum radial preload is 50N, allowing observation of its changes within the required stress range of the material after exceeding 33N-40N.

Figure 24 reveals consistent trends among the collision fitting curve, impact fitting curve, and vibration fitting curve across the theoretical, simulation, and experimental stages. Furthermore, the fitted impact and vibration values decrease as the collision diminishes during the complete motion of the variable diameter internal drive device inside the space sleeve. After the third inter-stage transition, these values gradually approach zero, signifying a smooth and uninterrupted contact between the elastic wheel and the inner wall of the space sleeve.

In Fig. 24a, the collision peak occurs at the first inter-stage transition before 2 s, whereas in Fig. 24b, the impact peak appears at 2 s due to the internal deformation of the elastomer impeding the transmission of the stress wave. Additionally, Fig. 24c demonstrates that the vibration peak occurs both before and after 2 s. This is because the sole force restraining the contact between the elastic wheel and the discontinuous surface is the radial preload, resulting in vibration during collision in the preload assembly, and another peak emerges after the impact as the stress wave transforms into a vibration wave.

From three perspectives—namely, theoretical analysis, numerical simulation, and experimental verification—it is evident that when the variable-diameter internal drive device operates within the sleeve, it encounters scenarios such as the gradual change in sleeve diameter and the presence of discontinuous surfaces between each stage. If the elastic wheel fails to make contact with the rigid surface after increasing the motion speed (enhancing the deployment efficiency of the space telescope), it will adversely affect the gradual deployment of the space sleeve. Ultimately, the space telescope may be unable to successfully complete deployment. To ensure effective contact between elastic wheels and non-continuous rigid surfaces, it is essential to analyze the relationship between motion speed, radial preload, and contact gap response values. Through mutual verification from three aspects, it becomes apparent that within the same grade of sleeve diameter, under the condition of ensuring the same radial preload, greater movement speed results in a larger collision impact and a larger contact gap. Similarly, under the same motion speed, a larger radial preload leads to a smaller collision impact and a smaller contact gap. The comparative analysis of the three aspects also clarifies that if the motion speed of each sleeve diameter stage is consistent, the output value of the radial preload needs to be adjusted accordingly; otherwise, the opposite effect may occur. Therefore, to ensure effective contact between the elastic body and discontinuous surfaces, it is necessary to timely adjust the motion speed and radial preload at each stage. This adjustment aims to reduce the vibration amplitude response generated by the process of "elastic collision-induced impact stress, impact stress transformation into stress waves, and stress wave transformation into vibration waves," consequently minimizing the vibration gap. In other words, the strategy is to leverage the frequency of external forces and motion velocity to prevent the resonance frequency between the elastic body and the discontinuous rigid body surface from approaching. As the frequency approaches, resonance can occur between the two, resulting in maximum vibration response and particularly intense amplitude changes. Therefore, active intervention to influence the contact frequency between the two, reduce vibration response, and alter amplitude changes is crucial for ultimately enhancing the stability of the variable-diameter internal drive device during the stage transition phase.

In summary, the three stages reveal important findings. Firstly, higher speeds lead to more significant changes in vibration amplitude caused by collision impact, resulting in larger contact gaps between the elastomer and the rigid body. Secondly, under constant speed conditions, higher preload forces result in smaller changes in vibration amplitude and reduced contact gaps between the two. Therefore, to ensure the efficiency of the variable diameter internal drive device during stage transitions, it is necessary to increase the radial preload force at the transition points while raising the operating speed. This adjustment ensures that the contact gaps during the transition are consistent with those at the continuous surface stage, allowing the elastomer to maintain stability on the discontinuous surface.

## Conclusions

The application of the reducer internal drive in space split telescope deployable mechanisms effectively addresses the limitations of traditional mechanisms concerning deployable distance and stiffness. However, when high-friction elastomers are used as the driving contact part in the reducer internal drive within the unique characteristics of the sleeve interior, increasing the operating speed of the reducer internal drive leads to elastic collision impact between the elastomer and the rigid body (non-continuous step surface). This, in turn, generates elastomer vibration and affects the contact state, thus reducing stability during stage transitions of the reducer internal drive. Therefore, this paper analyzes and establishes the relationship between elastomer velocity and radial preload on the elastic contact state during inter-stage transitions based on elastic collision impact theory. It is verified that the speed of motion and radial preload can regulate the elastic contact state, ensuring stability during transitions and promoting the effective deployment of the space expandable mechanism. The highlights of this work are as follows:

1. Analyze the motion state of an elastic body on a transitional rigid discontinuous surface, and establish models for elastic collisions, impacts, and vibrations.
2. Utilize the constitutive model of rubber elastic materials to derive collision models under various velocities and radial preloads. Calculate positive and negative waves generated during impact conditions, and use them as external excitation to determine vibration amplitudes.
3. It was discovered that motion speed significantly influences the elastic contact state between the elastic body and the rigid body. Adjusting the radial preload effectively controls the elastic contact gap, enabling the variable diameter internal drive device to increase speed while maintaining stability during stage transitions.
4. Employ finite element analysis to assess the impact of different velocities and radial preloads on the elastic contact state. Fit curves depicting the variation of the elastic body's vibration amplitude under varying velocities and radial preloads during stage transitions.
5. A 1:1 experimental prototype was designed and manufactured to validate the relationship between motion speed, radial preload, and elastic body vibration amplitude, both of which have a significant impact.

Due to limitations in knowledge and time, this study primarily focused on theoretical research and experimental verification of the elastic contact characteristics between elastic and rigid bodies during the inter-stage transition of a variable diameter internal drive device within a deployable mechanism in space. Nevertheless, this work is preliminary, and the scope of future research is suggested as follows: In the investigation of contact characteristics between elastic and rigid bodies during the stage transition, only the effects of changes in velocity and preload on the contact state were considered. However, it remains to be explored whether alterations in the shape and size of micro-convex bodies on the surface of the elastic material could further reduce the energy generated by collision and impact, thus minimizing vibration amplitude. Therefore, future research will involve a more comprehensive analysis.

## Data availability Statement

The datasets used and/or analysed during the current study available from the corresponding author on reasonable request.

## Competitive interests

The authors declare that they have no knowledge of competing financial interests or personal relationships that may have influenced the work reported herein.

## Replication of results

The simulation data and theoretical model analysis data involved in this paper can be provided as required.

## References

[1] Thornton C, Cummins SJ, Cleary PW (2011). An investigation of the comparative behaviour of alternative contact force models during elastic collisions. Powder Technol..

[2] Gugan D (2000). Inelastic collision and the Hertz theory of impact. Am. J. Phys..

[3] Stevens AB, Hrenya CM (2005). Comparison of soft-sphere models to measurements of collision properties during normal impacts. Powder Technol..

[4] Zhou Y (2011). A theoretical model of collision between soft-spheres with Hertz elastic loading and nonlinear plastic unloading. Theoret. Appl. Mech. Lett..

[5] Rossikhin YA, Shitikova MV, Manh DT (2016). Modelling of the collision of two viscoelastic spherical shells. Mech. Time-Dependent Mater..

[6] Morro A (2016). Modelling of viscoelastic materials and creep behaviour. Meccanica.

[7] Rossikhin YA, Shitikova MV, Trung PT (2017). Analysis of the viscoelastic sphere impact against a viscoelastic Uflyand-Mindlin plate considering the extension of its middle surface. Shock Vib..

[8] Xu H, Jiang X (2017). Creep constitutive models for viscoelastic materials based on fractional derivatives. Comput. Math. Appl..

[9] Tayeb A, Arfaoui M, Zine A, Hamdi A, Benabdallah J, Ichchou M (2017). On the nonlinear viscoelastic behavior of rubber-like materials: Constitutive description and identification. Int. J. Mech. Sci..

[10] Svanadze MM (2018). On the solutions of quasi-static and steady vibrations equations in the theory of viscoelasticity for materials with double porosity. Trans. A. Razmadze Math. Inst..

[11] Dongmei Z, Shiqiao G, Shaohua N, Haipeng L (2017). Study on collision of threaded connection during impact. Int. J. Impact Eng..

[12] Khodadadi A, Liaghat G, Ahmadi H, Bahramian AR, Anani Y, Razmkhah O, Asemeni S (2019). Numerical and experimental study of impact on hyperelastic rubber panels. Iran. Polym. J..

[13] Rabbi MF, Chalivendra VB (2019). Mathematical modeling of viscoelastic material under impact load. J. Strain Anal. Eng. Des..

[14] Springhetti R, Selyutina NS (2017). Viscoelastic modeling of articular cartilage under impact loading. Meccanica.

[15] Alaci S, Filote C, Ciornei FC, Grosu OV, Raboaca MS (2021). An analytical solution for non-linear viscoelastic impact. Mathematics.

[16] Amabili M (2016). Nonlinear vibrations of viscoelastic rectangular plates. J. Sound Vib..

[17] Papangelo A, Putignano C, Hoffmann N (2020). Self-excited vibrations due to viscoelastic interactions. Mech. Syst. Signal Process..

[18] Wielentejczyk P, Lewandowski R (2017). Geometrically nonlinear, steady state vibration of viscoelastic beams. Int. J. Non-Linear Mech..

[19] Loghman E, Kamali A, Bakhtiari-Nejad F, Abbaszadeh M (2021). Nonlinear free and forced vibrations of fractional modeled viscoelastic FGM micro-beam. Appl. Math. Model..

[20] Lewandowski R, Wielentejczyk P (2017). Nonlinear vibration of viscoelastic beams described using fractional order derivatives. J. Sound Vib..

[21] Guan Y, Huang W, Wang H, Lu H, Yang H (2023). Research on frictional characteristics of a space split telescope deployable mechanism variable diameter internal drive device in the inter-stage transition phase. Meccanica.

[22] Huang W, Guan Y, Wang H, Lu H, Yang H (2023). Research on the dynamics of the space tubular expandable structure driving deployment unit. Appl. Sci.-Basel.

[23] Zhang, J, *A Collision Force Model Based on Viscoelastic Theory and Its Application in Collision TMD*. Harbin Engineering University; 2018.CNKI:CDMD:2.1018.081400.

[24] Gladwell GML (1980). Contact Problems in the Classical Theory of Elasticity.

